# Application of self-supervised approaches to the classification of X-ray diffraction spectra during phase transitions

**DOI:** 10.1038/s41598-023-36456-y

**Published:** 2023-06-09

**Authors:** Yue Sun, Sandor Brockhauser, Péter Hegedűs, Christian Plückthun, Luca Gelisio, Danilo Enoque Ferreira de Lima

**Affiliations:** 1 Software Engineering Department, Institute of Informatics, University of Szeged, Dugonics tér 13, Szeged, 6720 Hungary; 2 European XFEL GmbH, Holzkoppel 4, 22869 Schenefeld, Germany; 3 Center for Materials Science Data, Humboldt-Universität zu Berlin, Zum Großen Windkanal 2, 12489 Berlin, Germany; 4 Deutsches Elektronen-Synchrotron (DESY), 22607 Hamburg, Germany

**Keywords:** Computer science, Phase transitions and critical phenomena

## Abstract

Spectroscopy and X-ray diffraction techniques encode ample information on investigated samples. The ability of rapidly and accurately extracting these enhances the means to steer the experiment, as well as the understanding of the underlying processes governing the experiment. It improves the efficiency of the experiment, and maximizes the scientific outcome. To address this, we introduce and validate three frameworks based on self-supervised learning which are capable of classifying 1D spectral curves using data transformations preserving the scientific content and only a small amount of data labeled by domain experts. In particular, in this work we focus on the identification of phase transitions in samples investigated by x-ray powder diffraction. We demonstrate that the three frameworks, based either on relational reasoning, contrastive learning, or a combination of the two, are capable of accurately identifying phase transitions. Furthermore, we discuss in detail the selection of data augmentation techniques, crucial to ensure that scientifically meaningful information is retained.

## Introduction

Experimental techniques such as spectroscopy and x-ray diffraction are instrumental in investigating matter (see, e.g., Ref.^1–4^). When experiments are performed at modern x-ray facilities, such as synchrotron radiation sources, and x-ray free electron lasers (XFELs), a vast amount of data are potentially collected over short periods of time. The ability to rapidly and accurately assess the status of an experiment is essential to maximize its efficiency. As an example, one may want to rapidly identify structural variations in a sample as a function of some external variables, or to monitor the sample damage due to X-rays. On the other hand, when analyzing data already collected—potentially up to hundreds of thousands of data sets—it is crucial to be able to employ some automated or semi-automated methods capable of extracting scientifically interesting features in the data so to minimize the usage of experts’ time and to maximize the scientific output.

Methods based on machine learning (ML) are ideal for automation of repetitive tasks and identification of features and patterns in data sets, and several applications to data collected at x-ray facilities have been recently published (see, e.g., Ref.^8–11^). In general, scientists have two tools at their disposal: (i) clustering the data to distinguish between different classes of samples, or (ii) labelling selected data to train a supervised classifier. When considering 1D spectral data, numerous clustering methods are useful at an exploratory stage, such as Spectral Clustering^11^, K-Means^13^, Agglomerative Clustering^14^, DBSCAN^15^. However, one major limitation of unsupervised clustering algorithms is that it can be challenging to determine the appropriate number or density of clusters to be discovered^16–18^. This often requires fine-tuning of certain hyperparameters to obtain accurate results. Following the exploratory stage, classification methods, including k-nearest neighbors^19^, partial least squares discriminant analysis^20,21^, decision trees^22^, random forests^23^, and extreme learning machines^24,25^, are typically employed to label the data. These traditional supervised ML models offer increased accuracy, but they rely on extensive and time-consuming process of labelling data. While traditional supervised ML models can achieve better performance, they heavily rely on hand-crafted features, thereby hindering the automated data analysis and limiting feature representation capabilities. Current popular methods are based on deep neural networks (DL), of which the most commonly established are convolutional neural network (CNN)^26–28^, recurrent neural networks (RNNs)^29,30^, attention-based neural networks^31,32^, and hybrid models^30,31,33^. They enable end-to-end learning of feature representations directly from raw data, and can scale effectively to large and complex datasets. However, it should be noted that the strength of supervised ML methods, that is the possibility of introducing domain-knowledge through annotation, is often problem-specific and time-consuming, which again hinders automation. Recently, methods based on self-supervised learning have opened up a new research frontier^34^. These are based on data augmentation techniques and appropriate pretext tasks, through which deep neural networks can learn generalizable features from unlabeled data. Self-supervised methods aim to establish a map from the data samples to a vector representation that summarizes the relevant information in the data. Ideally, one would like to produce a representation that is similar between two samples if they differ slightly in scientific content. For instance, if two samples of the data contain the same scientific information, but different levels of noise, one would expect them to be represented by similar vectors. This set of methods achieve that by requiring a neural network to solve "pretext" tasks, on which features are compared with transformed versions of the input data. The transformation may consist of, for instance, adding noise to the data. Such a pretext task would require the vector representations to be similar if the original data inputs are similar, but allow for different representations if the input data differs. The transformations used are referred to as augmentations. Given the critical role of the augmentations, it is important to select them wisely in a way that fit the problem at hand^35–38^. After building general representations of data, these may be more easily classified using a simple linear classifier, which would take advantage of the patterns discovered as part of the self-supervised learning stage. While self-supervised learning requires domain-specific knowledge, the need for human supervision is largely reduced with respect to supervised learning and the potential for automation is increased. In this study, we focus on two branches of self-supervised learning, that is self-supervised relational reasoning learning^39–42^ and self-supervised contrastive learning^35,36,38^.

The relational reasoning networks are based on a key design principle, that is the use of a relation network (usually a multi-layer perception, MLP) as a learnable function to quantify the relations between entities and their properties^40^. While the relational reasoning paradigm has gained traction in the deep learning community only recently^40^, it has achieved promising results in many fields, for example, video processing^41^, few-shot natural image recognition^42^, and time series data classification^39^. However, its application in the natural sciences is still scarce. Contrastive learning^43^ is based on learning similar/dissimilar representations from unlabeled data. The key principle is to extract underlying patterns in data by maximizing similarities of augmentations from the same instances while minimizing the similarity of different instances^35^. Recently, contrastive learning has attracted increasing attention in the natural sciences and has shown remarkable results on a variety of scientific problems, including molecular representation^44,45^, prediction of density-of-states of 2D photonic crystals^46^, similarity search for sky surveys^47^, single-particle diffraction images^48^. In particular, Ref.^46^ shows that self-supervised contrast learning can greatly reduce the number of labels required to train a network, which is a tedious and time-consuming operation. These successful applications in different scientific fields demonstrate the effectiveness and versatility of contrastive learning.

In this work, we demonstrate that self-supervised machine learning methods can provide great opportunities to improve the scientific efficiency of experiments at large-scale x-ray facilities. We explore the application of self-supervised relational reasoning and contrastive learning to 1D spectral classification problems. In particular, we show that it can be effectively used to classify phase transitions observed in X-ray diffraction (XRD) experiments^49–51^. We introduce and discuss three self-supervised representation learning frameworks for the classification of data, namely SpecRR-Net, SpecMoco-Net, and SpecRRMoco-Net. SpecRR-Net extracts discriminative features from unlabeled spectra based on relational reasoning, which attempts to discover data representations by reasoning the relation among entities^39,40^ in multiple dimensions and at different scales. SpecMoco-Net is based on contrastive learning, which aims to build representations by learning similarities and dissimilarities between different objects^35,36^. SpecRRMoco-Net benefits from both relational reasoning and contrastive learning, and combines SpecRR-Net and SpecMoco-Net. The backbone encoders applied in all three models were adapted from the ConvSC attention model in Ref.^31^, which was specifically designed for 1D spectral classification. We furthermore demonstrate the validity and performances of these three frameworks targeting the identification of a phase transition as seen by x-ray diffraction. The results show that the methods can effectively reduce the time spent by scientists annotating data manually, therefore offering great potential to automate the classification process.

## Methods

In this section, after introducing the case study, we present the proposed self-supervised spectral classification framework, shown in Fig. 3. It includes self-supervised pre-training to learn useful representations from unlabeled spectral data, and downstream supervised classification based on small amounts of labeled data. Self-supervised learning methods generally include two aspects: pretext tasks and loss functions. A crucial step for the success of these methods is the definition of proper objectives for unlabeled data in conjunction with data augmentation. In this work, we define four pretext tasks by exploring the meaningful information of 1D spectral data itself. Based on this, four surrogate-objective functions are proposed. In this way, useful representations can be learned by solving these pretext tasks, with the aim of significantly reducing the number of labels and increasing the automation of the classification process. In the following, we first describe the use case, then introduce the formulation of the problem and detail the data augmentation applied in this work, and finally discuss our approach.

### Experimental data

**Figure 1:**
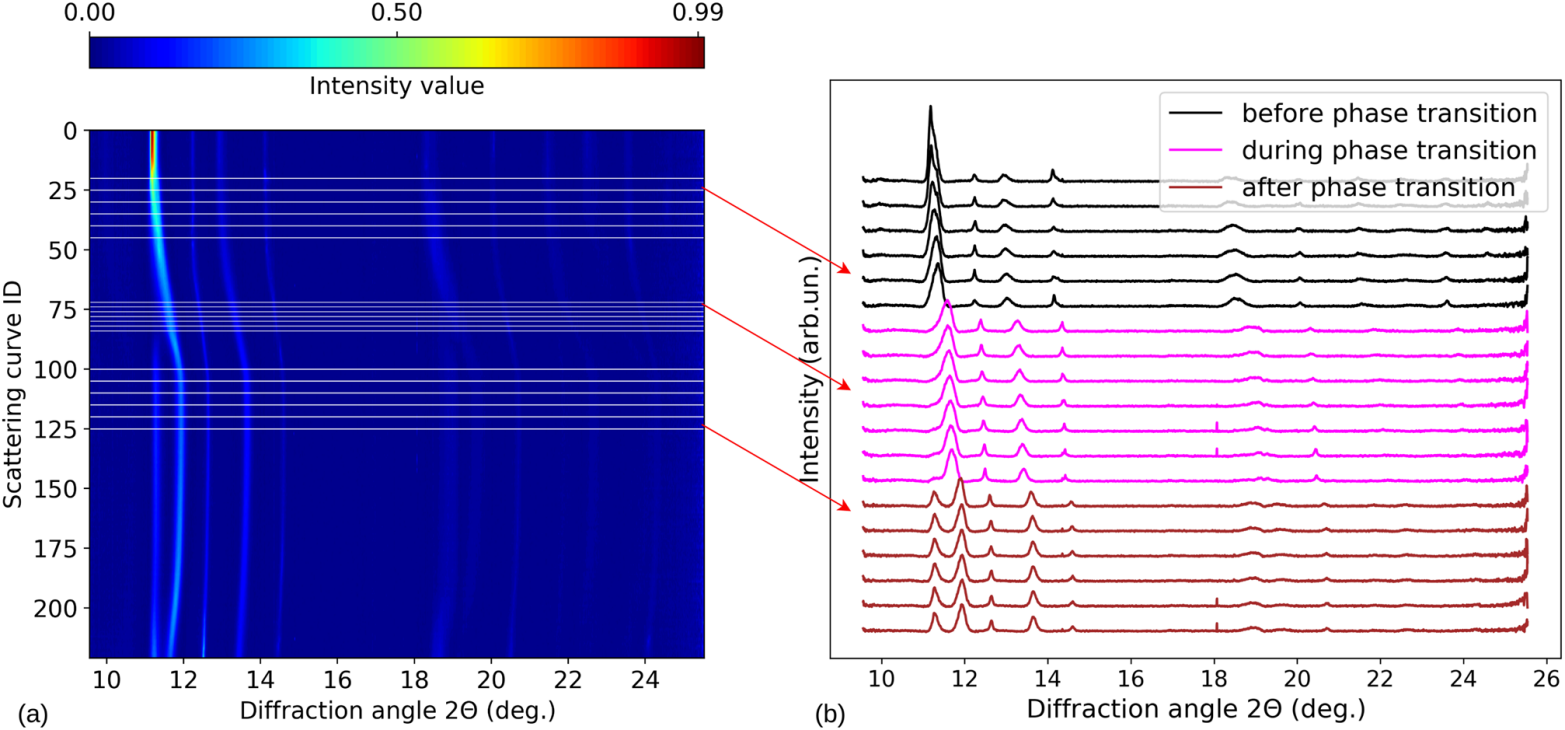
X-ray scattering curves corresponding to dataset D8 (wüstite powder sample). (**a**) Intensity distribution for different curves collected applying different pressures drawn as contour plot. The horizontal lines correspond to representative curves shown in (**b**). Here, the color black corresponds to the original B1 structure (that is, before the phase transition), magenta to the transition, and brown to the rB1 structure (that is, after the phase transition). In (**b**) curves are shifted vertically to improve visualization.

To validate the proposed methods based on self-supervised learning, we employ experimental x-ray powder diffraction data collected^52,53^ by applying different pressures to iron (Fe) and wüstite (FeO) samples^53^. Examples of scattering curves are shown in Fig. 1. For both, different crystal structures (allotropes) are thermodynamically stable within different pressure ranges. In particular, for iron a transition from body-centered cubic (BCC) to Hexagonal Close-Packed (HCP) is expected above approximately 13 GPa^53,54^. In the momentum transfer range accessible to our experiment, there are 3 Bragg reflections for the BCC, and 6 for the HCP structure. Bragg peaks are expected to change in position as a function of pressure, and potentially in profile, reflecting inhomogeneous strains and various kind of defects (see, e.g., Ref.^55^). In this case, the task set for our self-supervised methods is to detect the BCC-HCP phase transition, and in particular to identify patterns corresponding to either the BCC or HCP atomic arrangement which are characterized by different Bragg peaks, or the region corresponding to the transition between the two. Seven different data sets were collected, each one characterized by the application of pressure at different rates or signal-to-noise ratio (individual data sets are labeled as D1 to D7). Further details for each collected dataset, including the number of scattering curves corresponding to before (BCC), during and after (HCP) the phase transition are shown in Table 1. To simplify the presentation of results, they are all summarized as data set “Fe” in later sections. In the case of wüstite, the target is the identification of the B1 to rB1 crystal structure transformation above approximately 14–15 GPa^53,56^. Also in this case, two data sets were collected by applying pressures at different rates (D8 and D9, details in Table 1). To simplify presentation, they are summarized as “FeO”. Data sets D1 to D9 are composed by a different number of scattering curves, 60 to 460 (see Table 1), each containing approximately 4,000 data points (features). The number of labels annotated for each class is also reported in Table 1. Please notice that only a limited fraction of labels corresponding to the transition between two phases in FeO is available. All the data were collected on powder samples at the P02.2 beamline of the synchrotron light source PETRA III^57^ at DESY, at a photon energy of 25.6 keV^53^ and with two LAMBDA GaAs 2 M detectors^58^. The corresponding 1D diffraction spectra were then obtained by azimuthal integration and background subtraction^59,60^. Representative curves are shown in Fig. 1. Pressure, up to peak values of 65 GPa (Fe) and 46 GPa (FeO), was applied to the samples using either piezo actuator driven dynamic diamond anvil cell (dDAC)^61,62^ or the membrane diamond anvil cell (mDAC)^63^. Further details on the experiment are provided in Ref.^53^

**Table 1:** Information on the samples used in the X-ray powder diffraction experiments.

Dataset	Sample	Τ(ms)	Pْ (GPa/s)	N	B/D/A
D1	Fe	10	260	60	17/0/43
D2	Fe	100	10	116	29/0/87
D3	Fe	10	160	70	21/0/49
D4	Fe	1	1,030	130	33/0/97
D5	Fe	100	10	130	37/0/93
D6	^†^Fe	10	262	70	22/0/48
D7	^†‡^Fe	10	287	258	19/0/239
D8	FeO	50	7	222	71/20/131
D9	FeO	50	2.2	460	90/65/305

For every dataset, the sample is either composed of iron (Fe) or wüstite (FeO). The phase transition for all Fe and FeO samples is detected at a pressure of about 15 GPa. The compression rate (Pْ) is also reported, together with the detector exposure time (Τ). The last two columns show the total number of diffraction patterns collected for a given dataset (N), and the one corresponding to the three possible classes, as labeled by an expert. These are before (B), during (D) and after (A) phase transition, from the BCC to HCP structure for Fe, from B1 to rB1 for FeO. ^†^A pressure calibrant (platinum) was employed. ^‡^A pressure medium (neon) was employed.

### Problem definition

Given unlabeled data containing a series of spectral curves $\left\{ {x_{i} } \right\}$, we aim to learn a parametrized map $f_{\theta } \left( \cdot \right)$, which can produce a rich and descriptive representation $z_{i} = f_{\theta } \left( {x_{i} } \right)$ from unlabeled spectra for the downstream classification task. In this equation, θ are the learnable parameters of the neural networks. The learned representations will be then used for downstream spectral classification tasks while using a minimal number of labels.

### Data augmentation

Data augmentations, which provide different views of the input data expected to be mapped to similar representation vectors, are critical in defining useful pretext tasks^35^ in self-supervised learning. Such augmentations produce varied spectra, possibly with simulated additional experimental complexity or noise, but still plausible and with the same target labels. The objective function therefore ensures that same-label variations of the input spectra must be represented similarly. Such a procedure increases the robustness and generalization capabilities of the model, as variations of the input dataset are also used to train the model.

**Figure 2:**
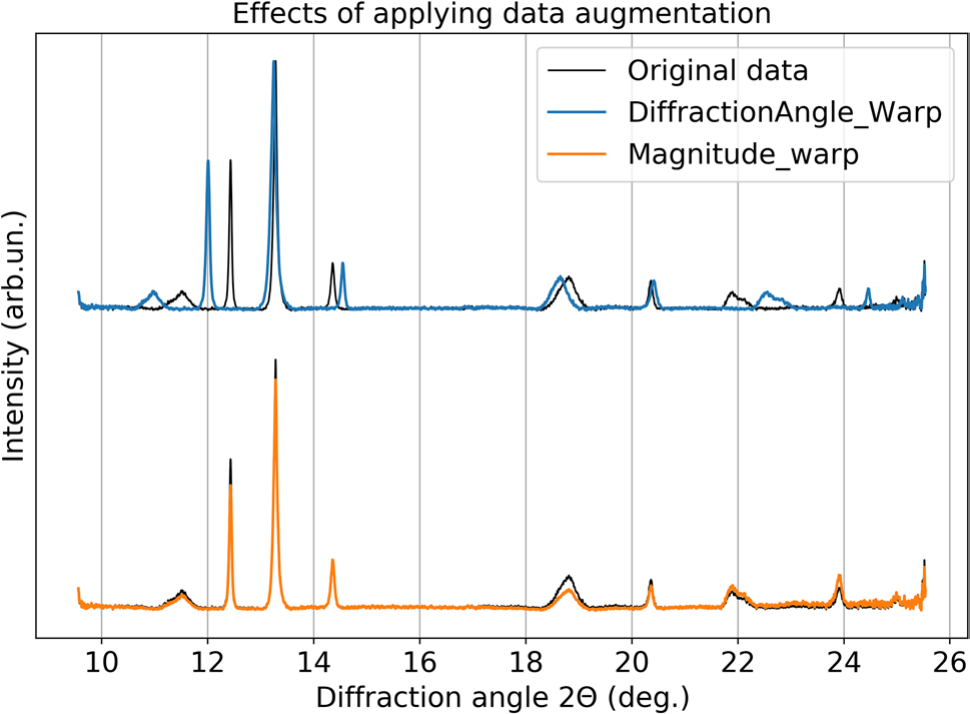
Example of 1D scattering curve, and the effect of applying magnitude warping and diffraction angle warping data augmentations to diffraction spectra.

In this work, we first preprocessed the spectra data by normalizing them to the [0, 1] range, then we sequentially applied diffraction angle warping (which is adapted from time warping^64^ changing its original time dimension to the diffraction angle dimension), and magnitude warping^64^ as data augmentations. Magnitude warping is used to simulate reasonable and random variations in the intensities of peaks, while not changing their positions. Diffraction angle warping is used to parallel the variation of peak positions, so to allow the model to focus more on the number of peaks rather than their location. An example of the effect of the augmentations is shown in Fig. 2. It is important to note that both data augmentations are physically meaningful and specific to the case of study. In fact, the application of neither data augmentation techniques results in changes in the number of the peaks or aspects relevant for the detection of a phase transition.

### Self-supervised pre-training and linear evaluation on downstream 1D spectra classification

**Figure 3:**
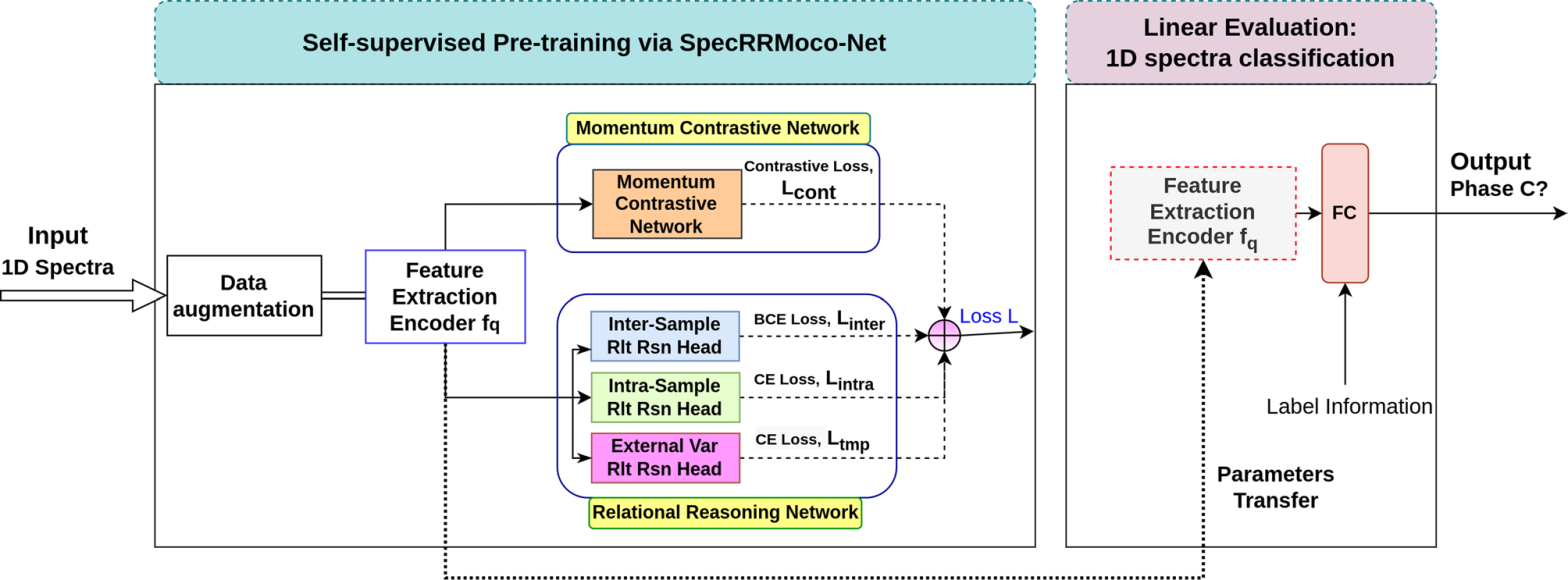
Illustration of the proposed 1D spectra classification framework based on the self-supervised SpecRRMoco-Net, which is a combination of Relational Reasoning Network (SpecRR-Net) and Momentum Contrast Network (SpecMoco-Net). The classification framework consists of two parts, namely pre-training and linear evaluation of downstream spectral classification. In the pre-training stage, the encoder $f_{q}$ is trained on unlabeled data to build useful representations; in the linear evaluation stage, a small number of labels are used to perform the downstream spectral classification task, where a linear classifier is trained on top of the frozen feature extraction encoder $f_{q}$. Specifically, the encoder is trained by jointly minimizing the contrastive loss $L_{cont}$ in SpecMoco-Net, inter-sample relational reasoning loss $L_{inter}$, the intra-sample relational reasoning loss $L_{intra}$, and the external variable relational reasoning loss $L_{tmp}$ in SpecRR-Net.

The self-supervised classification framework adopts a two-stage training, i.e., a pre-training stage and a linear evaluation stage, as shown in Fig. 3. In the pre-training stage, the feature extraction backbone encoder is trained in an unsupervised manner through momentum contrastive learning and relational reasoning-based learning. During this stage, the backbone encoder projects input data into a latent space $z$, which provide another representation of the data. As part of the training, a transformation $g\left( \cdot \right)$ is applied on the vector $z$, to obtain the output used in the loss function (defined below). Such transformation is referred to as ‘contrast head’ or ‘relational reasoning head’. The objective of this pre-training is to learn useful representations ${\text{z}}$ from the unlabeled spectra under the supervision of self-supervised pretext tasks, thus reducing the amount of label information needed for downstream classification task. After the pre-training, the contrast head and relational reasoning heads are discarded to reduce the correlation between output variables, as it has been suggested in related self-supervised learning research to learn better representations (see, e.g., Ref.^35,36,39^), the backbone parameters are completely transferred to the second part for downstream classification tasks. In the linear evaluation stage, the feature extractor is frozen, and a single-layer linear classifier is trained using a reduced amount of labeled data, projecting the learned representations in the latent space to physically meaningful spectral phase classes.

The shared feature extraction backbone model $f_{q}$ applied in this approach is the Conv SC attention model from Ref.^31^, but without the feed-forward network, as shown in Fig. S1 of the supplementary material. It consists of two convolution modules for extracting local features, and two self-attention modules performed across spatial (diffraction angle) and channel (introduced by the convolutional channels) dimensions to build long-range dependencies of spectra. In this way, latent dependencies and useful representations can be well captured. Furthermore, to accept input data with different feature sizes, we apply the 1D adaptive average pooling instead of the 1D global max pooling operation in the second convolution module, as shown in Fig. S1 of the supplementary material (see Ref.^31^ for more details on this model).

In this work, four pretext tasks are proposed to supervise the training of the backbone encoder in the pre-training stage. These include three relational reasoning-based pretext tasks, i.e., an inter-sample relational reasoning module, an intra-sample relational reasoning module, an external-variable relational reasoning module, and one pretext task based on instance-level contrastive learning, as shown in Fig. 3. We name the self-supervised classification framework based only on three relational reasoning modules as SpecRR-Net, the network based only on the contrastive module as SpecMoco-Net, and the combination of these two networks as SpecRRMoco-Net. We will describe each module in detail in the following sections.

### Inter-sample relational reasoning

**Figure 4:**
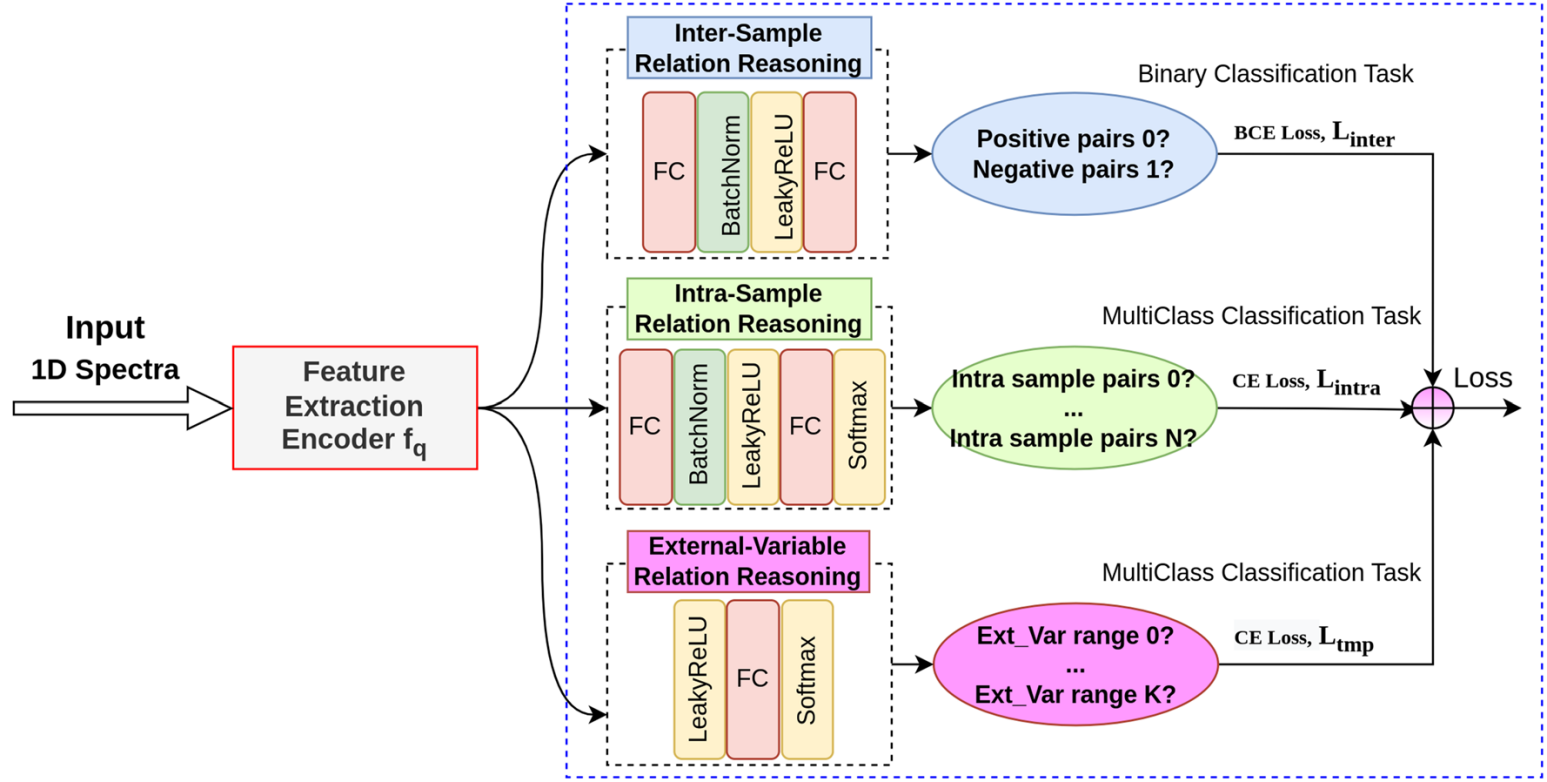
Illustration of the self-supervised relational reasoning sub-network (SpecRR-Net). It mainly consists of inter-sample, intra-sample, and external-variable relational reasoning modules. In this cartoon, $L_{inter}$ represents inter-sample relational reasoning loss, $L_{intra}$ represents intra-sample relational reasoning loss, $L_{tmp}$ represents the external variable relational reasoning loss.

The Inter-Sample relational reasoning module^39,40^ learns to quantify the relationships of the sampled pairs (how spectral instances are related to themselves and other instances), by formulating it as a binary classification pretext task, as shown in the upper branch of Fig. 4.

Formally, given any spectral curve $x_{i}$, K random augmentations are applied to it to generate an augmented set $A\left( {x_{i} } \right) = \left\{ {x_{i}^{\left( k \right)} } \right\}_{k = 0}^{K - 1}$, where $x_{i}^{\left( k \right)}$ is the k-th augmentation of $x_{i}$. For each augmented scattering curve, a representation can be extracted. The inter-sample relational reasoning module formulates a binary task by classifying pairs of augmented data as similar (positive) and other pairs, as dissimilar (negative). A positive pair is built by aggregating representations of augmented versions of the same spectra, while a negative pair is sampled from two randomly paired different spectra. By solving this pretext task, this module learns relationships between different spectral data. Further details may be found in the SM-1 of the Supplementary Material and in Ref.^39,40^

### Intra-sample relational reasoning

The Intra-Sample relational reasoning module^39^ models the relationship between different spectral pieces within each individual spectral curve. It is adapted from the intra-temporal relational reasoning module in Ref.^39^, originally proposed to model the global temporal dependencies of time series data. Here, we extend it to the diffraction angle dimension. We formulate the intra-sample relational reasoning module as a multi-class classification task trained with cross-entropy loss $L_{Intra}$, which follows the loss function of Eq. (2) in Ref.^39^. The hyperparameters are consistent with those of the module in Ref.^39^. In this way, the underlying dependencies along the diffraction angle dimension can be captured. Further details are given in the SM-1 of the supplementary material.

### External variable relational reasoning

Although the above two relational reasoning modules can learn latent discriminative features from sampled pairs, they do not properly utilize information on external variable applied to the samples. The external variables could be any variation imposed in the experiment, e.g., temperature, pressure, or electric field. In our specific use case, this is pressure, which varies with time during compression and decompression. Guided by this, we designed and introduced a third relational reasoning branch (see Figs. 3 and 4), that is the external variable relational reasoning module, to build robust external dependencies from the spectral samples. This can further enable the backbone to learn useful patterns along the external variable dimension.

Formally, given any spectral curve $x_{i}$ collected at time step $i$, the encoded representation of its augmented version $x_{i}^{A}$ is denoted by $z_{i} = f_{\theta } \left( {x_{i}^{A} } \right)$. A single layer external-variable relation projection head $r_{\gamma } \left( \cdot \right)$ is applied to reason the external variable relation score, denoted as $s_{i}^{tmp} = r_{\gamma } \left( {z_{i} } \right)$. First, the spectral curves are evenly divided into T = 5 external variable relation categories in order of acquisition time. Then, a multi-class classification pre-task is constructed and trained with the cross-entropy loss $L_{tmp}$ as

1 $$L_{tmp} = - \frac{1}{B}\mathop \sum \limits_{i = 0}^{B - 1} \log \frac{{\exp \left( {s_{{i,y_{i}^{tmp} }}^{tmp} } \right)}}{{{\Sigma }_{t = 0}^{T - 1} \exp \left( {s_{i,t}^{tmp} } \right)}},$$

where $y_{i}^{tmp}$ is the external variable category. The ablation study of hyperparameters T is left to future work.

### Self-supervised contrastive learning module for 1D spectra classification

In the self-supervised contrastive learning module, instance–wise contrastive learning^35,38^ is employed, where each spectra instance is treated as a distinct class of its own and a pretext classifier is trained to distinguish between individual instances^65^. SpecMoco-Net is based on momentum contrastive learning (MoCo)^38^. This is formulated as minimizing InfoNCE-based contrastive loss function $L_{cont}$ proposed by Ref.^35,38^. During training, the unsupervised contrastive loss brings spectra containing similar spectral peak features closer together in latent space, while spectra with different spectral features are pushed farther apart. Dissimilarities, within our case of study, are, e.g., different number of peaks, at different positions, or with different shapes. Further details are given in the SM-1 of the supplementary material.

### Self-supervised loss function

As can be seen from Fig. 3, the above four modules share the same backbone encoder $f_{q}$. The training of the shared feature extraction encoder can also be viewed as multi-task learning. By jointly optimizing the inter-sample, intra-sample and external variable relational reasoning objectives and the self-contrastive learning objective, the final training loss function of SpecRRMoco-Net is specified as

2 $$L = L_{inter} + L_{intra} + L_{tmp} + c \cdot L_{cont} .$$

Here $c$ is a coefficient to adjust the weight of the contrastive loss. Ablation studies on this coefficient are presented in ‘Experiments and Results’ section. It is important to note here that this loss function allows one to compare several configurations and combine their operation, except for the corner cases of $c = 0$ and $c = \infty$ (achieved by retaining the $L_{cont}$ item with a coefficient of 1, while excluding the other three relational reasoning-based loss items in the loss function). A value of $c$ set to 0 would correspond to SpecRR-Net, while setting it to a large value increases the relevance of SpecMoco-Net.

## Experiments and results

### Implementation details

The SpecRRMoco model, SpecRR-Net, and SpecMoco-Net were trained using PyTorch on a single NVIDIA A100-PCIE-40 GB. The self-supervised backbone encoder $f_{q} \left( \cdot \right)$ is trained by minimizing the proposed joint loss function Eq. (2) with a stochastic gradient descent (SGD) optimizer^66^. D1, D4, D8, and D9 data sets (872 spectral curves in total), were used to train the encoder network $f_{q} \left( \cdot \right)$ to learn feature representations during the pre-training (without label information). The batch size was set to 512, and the capacity of the queue of keys, a key parameter in the momentum contrastive learning module, has been set to 872 × 2 (that is, twice the total number of training spectral curves in the pre-training stage). The queue of keys keeps previous representations of the data during the training of the momentum contrastive learning module, to provide many negative samples for comparison (see Ref.^36,38^ for further information). Within the SpecRRMoco-Net framework, experiments were performed with a loss factor of ${\text{c}} = 0.01$, unless otherwise stated. We applied data augmentations randomly 6 times in the inter-sample relational reasoning branch. In the pre-training stage, the initial learning rate of the optimizer was set to 0.15, a linear warmup for the first 50 epochs (from a value of 0.02) followed by a cosine decay schedule was applied to adjust the learning rate during training, and the weight decay was set to 1 × 10^−4^. While the relation scores in relational reasoning modules are similarity-based, we formulate each relational reasoning pretext task as a classification task, so accuracy-based metrics can be applied to evaluate their performance on these pretext tasks.

In the second stage, the backbone encoder was fixed and a linear classifier was trained by minimizing the cross-entropy loss function. A SGD optimizer with a learning rate of 0.15, and weight decay of 1 × 10^−4^ was applied. To further prevent overfitting, both a train/validation and an early stopping strategy, which stops the training when the validation accuracy does not increase relatively to its previous best value for M = 20 steps, were employed to train the linear classifier. In order to evaluate the performance of the model, all data were labeled to calculate the weighted precision and recall. However, only 42 (2.8% of the datasets) of the labeled data were used to train the linear classifier in the linear evaluation stage. These representative scattering curves were selected from D1, D4, D5, D8 and D9 data sets as the basis of the training/validation dataset. Furthermore, of the 42 labeled data 15 belonged to the "before phase change" class, 25 to the "after phase change" class, and 5 to the "during phase change" class. The remaining 1,474 spectral curves (97.2% of the data) were used to test the performance of the backbone encoder and linear classifier. Further details are provided in Supplementary Material SM-2.

### Linear evaluation on downstream classification task

**Figure 5:**
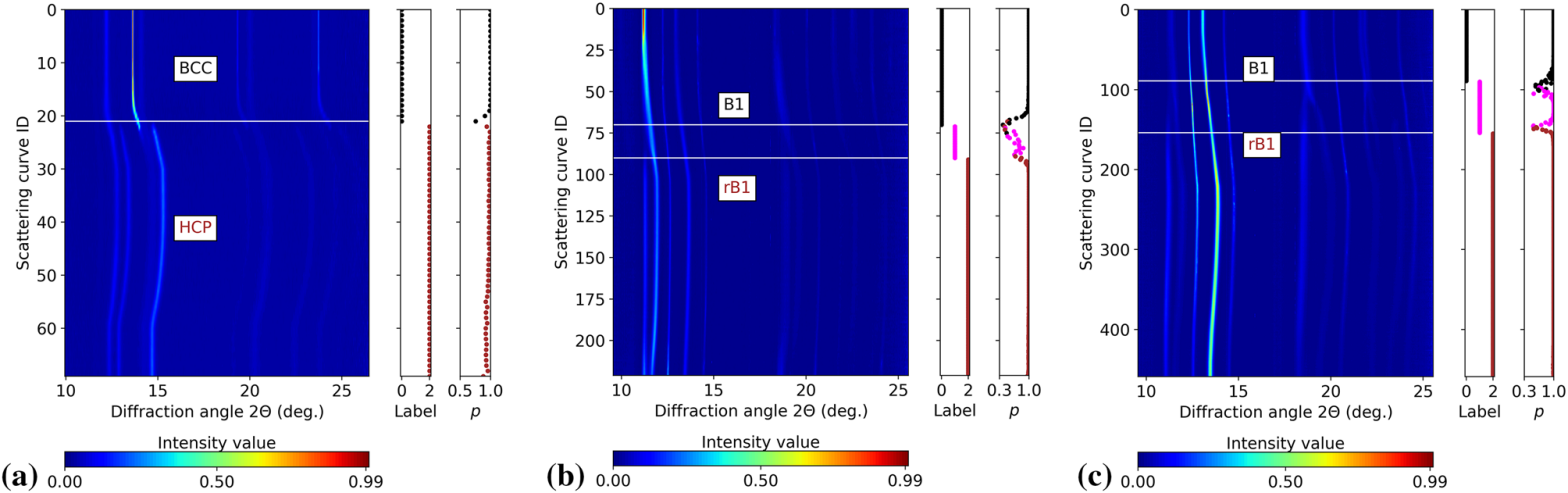
Classification results for experimental scattering curves using the proposed SpecRRMoco-Net with 2.8% labeled data. Each row of contour plots is a different scattering curve. The label as defined by an expert is also reported in the inset “Label”. Black corresponds to data sets collected before the phase transition (label 0, that is BCC for Fe and B1 for FeO), magenta during (label 1), and brown after (label 2, that is HCP for Fe and rB1 for FeO). Horizontal lines in contour plots indicate the onset or end of a phase transition. The label predicted by SpecRRMoco-Net is also reported (indicated by colors in the inset “p”), together with the associated probability $p$. Data sets shown are (**a**) D6, (**b**) D8 and (**c**) D9.

In this subsection, we evaluated the performance of the self-supervised encoder trained by different networks on the downstream spectral classification task. To do so, we train a linear classifier on top of learned representations from the frozen backbone encoder. As described above, 42 representative labeled spectra from five data sets were used to train the linear classifier. Figure 5 shows the classification results together with the classification probabilities of SpecRRMoco-Net for a few example data sets using only 2.8% of the labeled data. Specifically, the figure presents the results for D6 (Fig. 5a), D8 (Fig. 5b), and D9 (Fig. 5c). The classification results of the other 6 data sets are reported in Fig. S5 of SM-3 in the supplementary material. For each sub-figure, the first column renders the contour map of intensity distribution of the corresponding dataset, with the horizontal lines in the contour map indicating the phase transition boundary or phase transition interval. The second column shows the ground-truth labels of the dataset, where the black line represents class 0 (before phase transition), the magenta line represents class 1 (during phase transition), and the brown line represents class 2 (after phase transition). The last column shows the predicted category labels and the corresponding probabilities, where the values indicate the probabilities and the corresponding colors indicate the predicted labels. We compared the average classification precision/recall of SpecRRMoco-Net, SpecRR-Net, and SpecMoco-Net, as reported for Fe datasets (D1–D7) and FeO datasets (D8–D9) in Table 2. Average and standard deviation values were calculated from 20 runs. For each run, the results for the Fe data set or the FeO data set were obtained by averaging the results of all data sets within the group. The prediction time for each spectral curve is about 40 μs, which is small enough to meet the requirement of real-time processing even at high-repetition rate facilities like the European XFEL. While this procedure would not provide feedback to the users as soon as the experiment starts, since the network training creates a delay, after the network is trained with the first data, one may reuse the model for fast feedback for the rest of the experiment. The overall results show that with only 2.8% of the labels (42 spectral curves), all three models can accurately detect phase transitions in the Fe datasets, but some models do not perform well in the FeO datasets. In particular, SpecRRMoco-Net achieved better classification performance than SpecRR-Net and SpecMoco-Net under the current training strategy and hyperparameter settings, especially on FeO datasets, which are more challenging than Fe datasets due to the continuous nature of the phase transition and higher density of Bragg peaks. In SpecMoco-Net and SpecRR-Net (see also Fig. S6 of the supplementary material), the class labels of some spectra in D8 and D9 data sets were incorrectly predicted in the ‘during phase transition’ region. The classification results for the 10% labeled data are also reported in Table 2. A clear improvement of SpecRR-Net and SpecMoco-Net performances can be seen, with SpecRR-Net achieving a slightly better result than SpecMoco-Net. For SpecRRMoco-Net, good classification performance was achieved with 2.8% of labeled data, but the classification standard deviation on the FeO dataset is further reduced with 10% of labeled data. It should be noted that the other four data sets (D2, D3, D6, and D7 data sets) were not included in the training of the backbone encoder or linear classifier in the pre-training and linear evaluation phases, but nevertheless the self-supervised models still achieved very good classification results, meaning that the learned representation is transferable. Moreover, it also demonstrates the high quality of the learned representations of the feature extraction backbone encoder.

**Table 2:** Classification results measured in terms of weighted precision and recall using different self-supervised methods.

	Model	Fe		FeO	
		Precision	Recall	Precision	Recall
2.8% labels	SpecSelfTime	98.6 ± 0.2	98.5 ± 0.2	78.6 ± 3.2	80.0 ± 4.3
	SpecRR-Net	99.2 ± 0.3	99.1 ± 0.3	91.6 ± 6.2	90.7 ± 5.4
	SpecMoco	98.3 ± 1.1	97.9 ± 1.8	93.8 ± 3.7	93.2 ± 4.1
	SpecRRMoco	**99.6 ± 0.2**	**99.6 ± 0.2**	**96.3 ± 3.2**	**96.5 ± 2.4**
10% labels	SpecSelfTime	98.6 ± 0.5	98.5 ± 0.5	80.5 ± 2.7	82.0 ± 3.9
	SpecRR-Net	99.1 ± 0.2	99.0 ± 0.2	96.9 ± 0.8	95.8 ± 1.1
	SpecMoco	99.3 ± 0.3	99.3 ± 0.3	94.1 ± 0.7	93.7 ± 0.7
	SpecRRMoco	**99.6 ± 0.2**	**99.5 ± 0.2**	**97.1 ± 1.8**	**96.9 ± 1.3**

Best results are highlighted in bold.

For each method, the classification results are reported with amounts of labels corresponding to either 2.8% or 10% of the total collected data.

### Comparison with other methods

In this section, we compare the self-supervised classification models already introduced with a modified version of the SelfTime network^39^ (designed specifically for time series data), which we name SpecSelfTime. In particular, we replaced the original convolutional backbone encoder with the ConvSC attention network to better fit 1D spectral data for better performance. It should also be noted that SpecSelfTime, which is closely related to our work and the baseline of our SpecRR-Net, does not include the external variable relational reasoning module we introduced in this study.

For a fair comparison, the settings of hyperparameters in the SpecSelfTime model are the same as in the SpecRR-Net and SpecRRMoco-Net models. Table 2 show its classification results on experimental spectra with 2.8% labeled data (average weighted precision/recall 98.6/98.5% for Fe datasets and 78.6/80.0% for FeO datasets). In addition, for 10% of the labels, it does not have a great improvement in performance. SpecSelfTime performs poorly on several data sets, and particularly on FeO datasets, where it failed to detect the ‘during phase transition’ class on D8 (Fig. S6b of SM-3). This indicates poor generalization ability of the model. More classification results are presented in Fig. S6 of the supplementary material. As can be seen from the results, SpecSelfTime performs worse than the improved SpecRR-Net and even SpecMoco-Net, which highlights the importance of the external-variable relational reasoning module we introduced.

**Figure 6:**
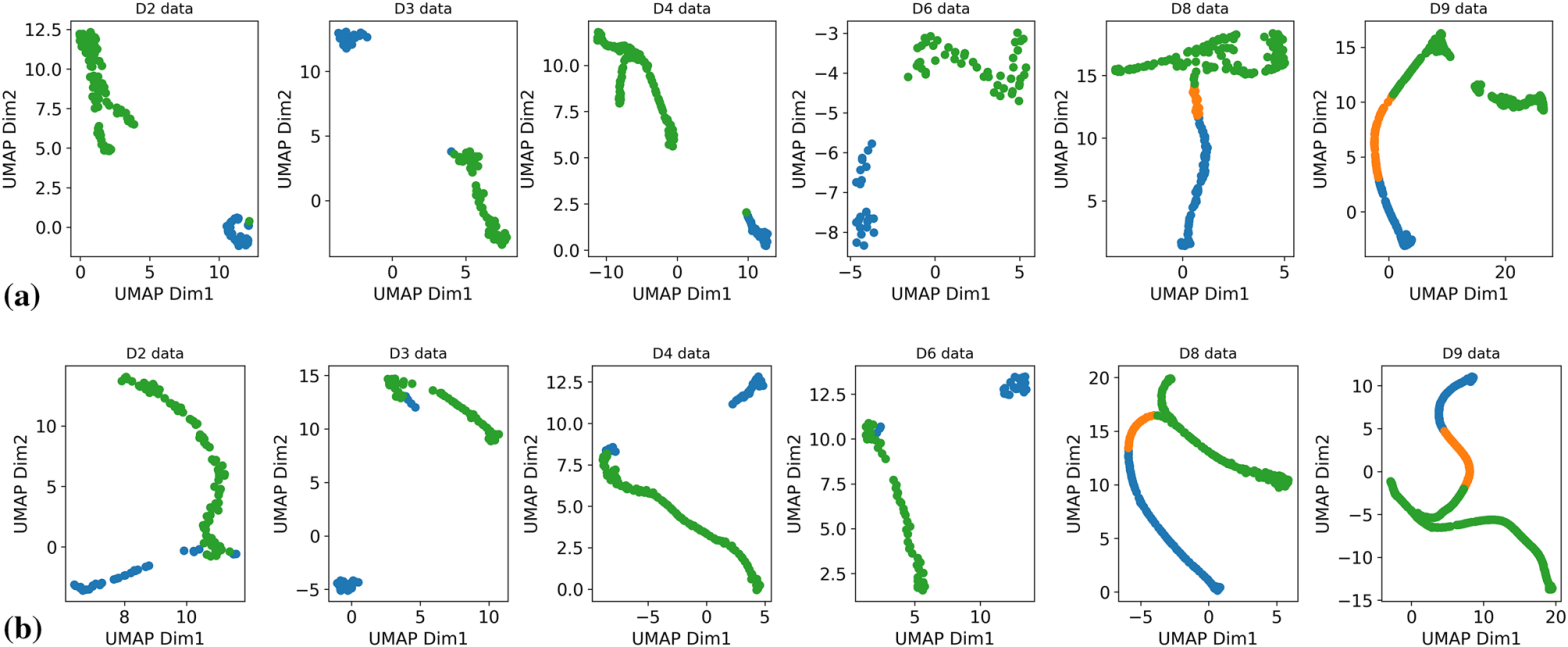
(**a**) UMAP visualization of the embedded features from some example datasets (D2, D3, D4, D6, D8, and D9, respectively) after the SpecRRMoco-Net encoder. (**b**) UMAP visualization of the original example data sets (D2, D3, D4, D6, D8, and D9, respectively). In both cases, the class labels are ground truth.

While the downstream classification task can evaluate the quality of the model, it cannot fully reflect the clustering ability. Therefore, as a qualitative analysis, we further evaluate the clustering power of these self-supervised classification models by visualizing the learned representations using UMAP (Uniform Manifold Approximation and Projection)^67^. Figure 6a renders the UMAP of SpecRRMoco-Net, while Fig. 6b visualizes the original data. In both cases, the class labels are ground truth. The UMAP visualization plots of SpecRR-Net, SpecMoco-Net, and SpecSelfTime are given in Fig. S7 of the supplementary material.

Often, there is the need to perform exploratory analysis on the acquired data, in order to understand patterns before attempting to perform any labeling. This procedure is different in scope from the direct classification and aims only at detecting similarities within the data. In this case, clustering methods are often used. For this reason, we compare, in Table 3, the capability of a traditionally used clustering method, Spectral Clustering, of finding similarities within each label either from the original data, or starting from the representation produced by the studied self-supervised methods. To assess the clustering quality, two metrics have been used: the Silhouette Score^68^ and the Mutual Information^69^ (the Rand score^70^ is also shown in the Supplementary Material Table S3). The Silhouette Score is shown in Table 3, and it measures how the distances between samples within a cluster compare with distances between clusters without using the ground truth label association. The Mutual Information Score is shown in Table 4, and it uses ground truth information and compares the mutual agreement in the assignment using information-theoretic approaches, while being invariant to permutations of the labels. These are calculated after clustering the data by applying Spectral Clustering to the learned representations of each self-supervised encoder, or by clustering the original data itself with Spectral Clustering. The effect of varying some hyperparameters of the Spectral Clustering may also be seen in the uncertainties, as the optimal choice may not be known during the exploratory analysis phase. In addition, in spectral clustering, a K-means strategy or Discretization strategy is applied to assign labels and the affinity matrix is constructed by computing the nearest neighbor graph or radial basis function (RBF) kernel. The mean and standard deviation values are calculated from different combinations of these parameters (four groups). As can be seen from the results, the representations learned by the relational reasoning self-supervised methods show better cluster separation ability compared to the original data. Changing the hyperparameter settings for both SpecMoco and the clustering of the original data may lead to different results, while the relational reasoning-based methods tend to lead to representations that are less dependent on choices of the hyperparameters in Spectral Clustering. Particularly, the choice of the label assignment in Spectral Clustering leads to a high variance in SpecMoco. On the other hand, combined with the previous linear evaluation results, SpecSelfTime shows poor classification performance while achieving relatively good clustering ability. This experiment shows that the representations learned from self-supervised methods may lead to good cluster separation ability without necessarily allowing for better classification performance within the scope of the linear evaluation protocol when only a small subset of labeled data is available for training.

**Table 3:** Average Silhouette coefficient obtained by applying different methods to each data set.

Model	Average	
	Fe	FeO
Spectral Clustering (SC)	0.64 ± 0.10	0.52 ± 0.07
SpecSelfTime + SC	0.87 ± 0.04	0.49 ± 0.06
SpecRRMoco (c 0.01) + SC	0.85 ± 0.02	0.57 ± 0.01
SpecRR-Net + SC	0.87 ± 0.00	0.61 ± 0.03
SpecMoco + SC	0.69 ± 0.15	0.42 ± 0.02

The first row shows the effect of applying Spectral Clustering directly to the data, while the following rows show the effect of applying Spectral Clustering to the latent representation produced after the encoder trained with the respective self-supervised learning techniques. Results for Fe datasets and FeO dataset are reported. In the spectral clustering, the number of clusters was set to 3 for the Fe data sets, and 2 for the FeO data sets. In addition, Spectral Clustering hyperparameters on the label assignment and on the method for building the affinity matrix have been varied, and the average result is shown with the root-mean-squared error over different configurations.

**Table 4:** Mutual information between the ground truth labels and the predicted labels of different methods for each dataset.

Model	Average	
	Fe	FeO
Spectral Clustering (SC)	0.36 ± 0.06	0.56 ± 0.02
SpecSelfTime + SC	0.49 ± 0.03	0.54 ± 0.07
SpecRRMoco (c 0.01) + SC	0.50 ± 0.03	0.64 ± 0.02
SpecRR-Net + SC	0.50 ± 0.01	0.64 ± 0.04
SpecMoco + SC	0.41 ± 0.12	0.63 ± 0.02

The first row corresponding to applying Spectral Clustering directly to the original data, while the following rows show the mutual information scores by applying Spectral Clustering to the latent representation produced after the encoder trained with the respective self-supervised learning techniques. Results for Fe datasets and FeO dataset are reported. Spectral Clustering hyperparameters on the label assignment and on the method for building the affinity matrix have been varied, and the average result is shown with the root-mean-squared error over different configurations.

### Ablation studies on the coefficient $c$ in the SpecRRMoco-Net loss function

Here, we report on an ablation study on the coefficient $c$ (shown in Table 4), performed to understand its impact on learning data representations. These experiments were performed under the same training setup described above. We varied $c$ in the range [0.001, 1], and also set it to 0 (that is, a pure SpecRR-Net) and infinity (that is, a pure SpecMoco-Net). For the downstream spectral classification task, 2.8% of labels were used. From Table 5, we can see that SpecRRMoco-Net performs well over a wide range of the coefficient $c$ (0.001–1 and infinity). This result suggests that jointly optimizing the relational reasoning-based pretext task and the contrastive learning-based pretext task can improve the performance of the pure contrastive learning-based network as well as the purely self-supervised relational reasoning network under the current training setup.

**Table 5:** Ablation study of the coefficient $c$ in the loss function.

c	Fe		FeO	
	Precision	Recall	Precision	Recall
0.001	98.5 ± 0.6	98.4** ± **0.4	92.7 ± 4.0	92.8 ± 3.3
0.01	**99.6 ± 0.2**	**99.6 ± 0.2**	**96.3 ± 3.2**	**96.5 ± 2.4**
0.1	**99.5 ± 0.2**	**99.2 ± 0.8**	**97.3 ± 1.0**	**97.0 ± 1.3**
1	98.9 ± 0.6	97.9 ± 1.6	95.2 ± 2.0	95.0 ± 1.4
0 (SpecRR-Net)	99.2 ± 0.3	99.1 ± 0.3	91.6 ± 6.2	90.7 ± 5.4
Inf (SpecMoco-Net)	98.3 ± 1.1	97.9 ± 1.8	93.8 ± 3.7	93.2 ± 4.1

The best values within uncertainties are highlighted in bold.

Weighted precision and recall for Fe and FeO are reported as average and standard deviations over 20 runs.

### Ablation studies on the data augmentation

We report here on an ablation study on data augmentations performed in order to evaluate their impact on the SpecRRMoco-Net performances. Several commonly used data augmentation techniques were explored, including diffraction angle warping (D.A.W.), magnitude warping (M.W.), window slicing (W.S.), jittering (Jitter), and scaling. Among them, diffraction angle and window slicing are performed in the diffraction angle dimension, whereas jittering, scaling, and magnitude warping are performed in the magnitude domain. The variations introduced by these data augmentation techniques respect physical information contained in the data itself, with effects that resemble the realistic range of experimental effects, without changing the data labels. They generate new input with variations while keeping identical labels in the embedding space. Based on this, surrogate tasks can be formed to extract underlying patterns and build the representations.

**Figure 7:**
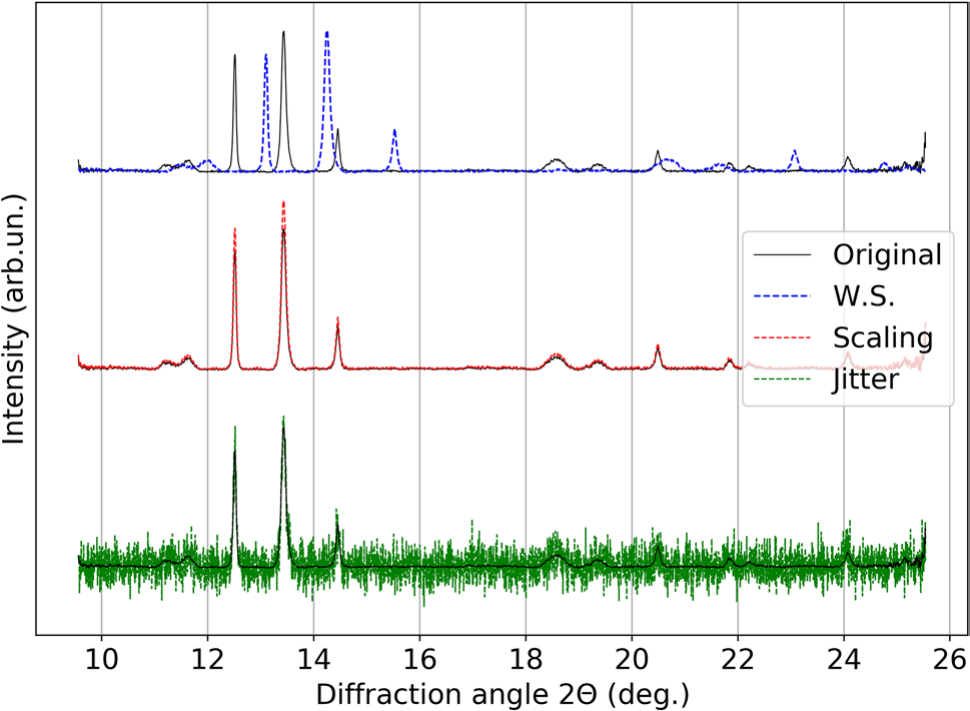
Illustration of the effect of applying window slicing (W.S.), scaling, and jittering (Jitter) data augmentation techniques to one of the scattering curves.

In addition to the diffraction angle and magnitude warping which were already discussed, jittering was used to introduce possible random noise in the experiment, such as additive detector noise. It was simulated by adding noise sampled from a Normal distribution with a mean value of 0 and a standard deviation of 0.1. Scaling was used to model uniform intensity variations, which is achieved by multiplying the original data by a random scalar value sampled from a Normal distribution with a mean of 1 and standard deviation of 0.1. Window slicing was used to model small variations in diffraction angle coverage, for example, when the sample and detector are far apart, resulting the detector covering a smaller range of diffraction angles. This is achieved by randomly cropping out a large continuous slice of the spectrum (in the implementation 80% of the original spectral length, i.e., randomly discarding 20% of the edge spectral segments) and interpolating it to the original length. An illustration of these three data augmentation techniques can be seen in Fig. 7. Scaling is used to simulate reasonable and random variations in the intensities of peaks, while not changing their positions. Diffraction angle warping is used to parallel the variation of peak positions, so as to allow the model to focus more on the number of peaks rather than their location. For further information on the parameters in the data augmentation techniques, see Supplementary Material SM-4.

**Figure 8:**
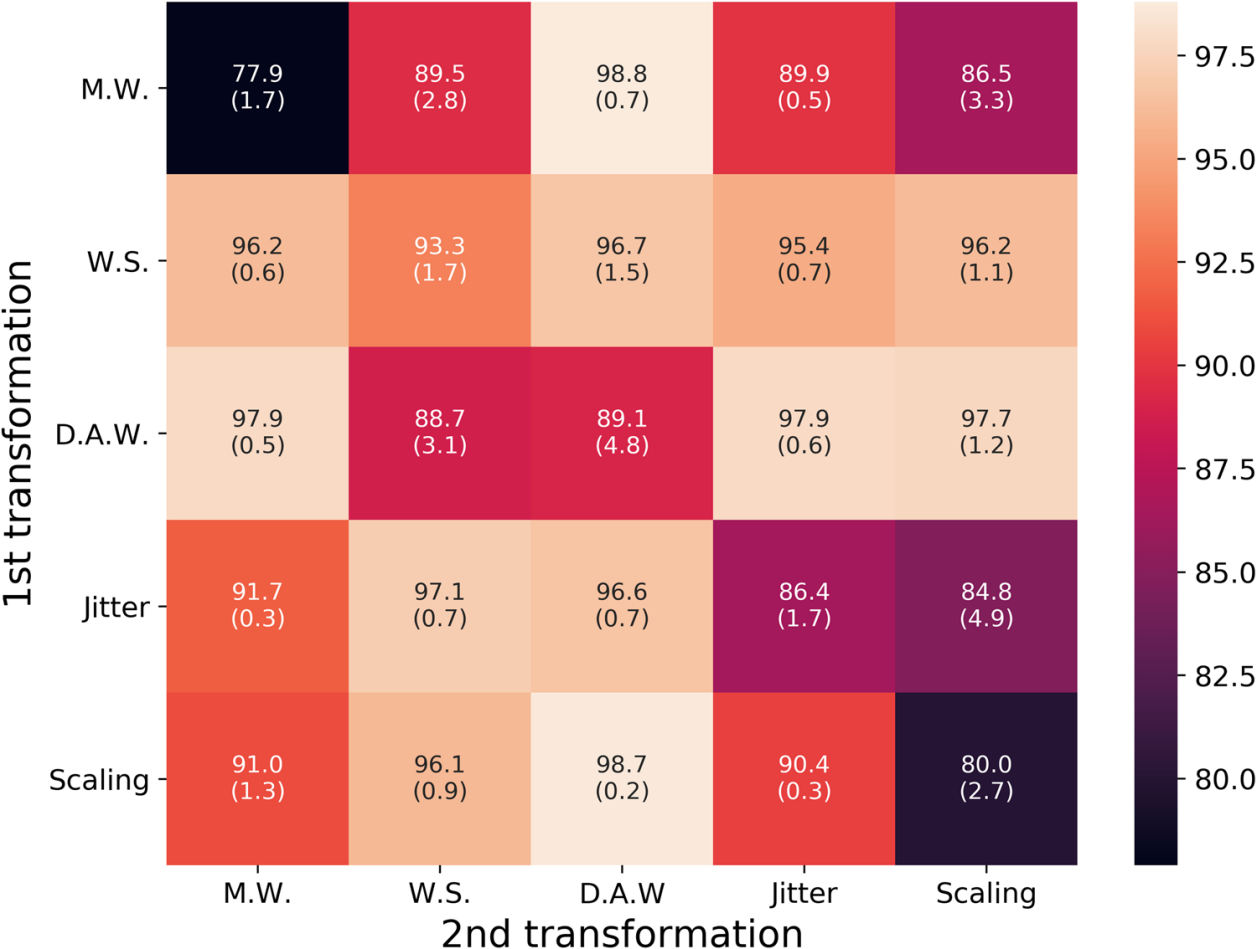
Ablation study on data augmentation techniques. Results for magnitude warping (M.W.), window slicing (W.S.), diffraction angle warping (D.A.W.), jittering (Jitter) and scaling data augmentation techniques are reported. The figure shows the average classification accuracy and standard deviation (values in parentheses) for 20 runs with 2.8% of labeled data. In addition to this, diagonal elements indicate the use of only one data augmentation technique, while other non-diagonal entries indicate the combination of two data augmentation techniques. The color scale represents the classification accuracy. This heatmap image was generated by the Python data visualization library seaborn^71–73^, version 0.10.0.

Figure 8 shows the linear evaluation (represented by the average accuracy and standard deviation of 20 runs for all nine datasets) under different data augmentation techniques individually or in combination. The diagonal elements correspond to a single data augmentation and the non-diagonal elements represents the combination of the two consequent data augmentation techniques. The classification results show that the combination of two data augmentation techniques usually performs better than a single technique. In particular, the best result is achieved when “magnitude warping” is combined to “diffraction angle warping”. Therefore, in this study we applied these sequentially to all models presented in this paper.

**Figure 9:**
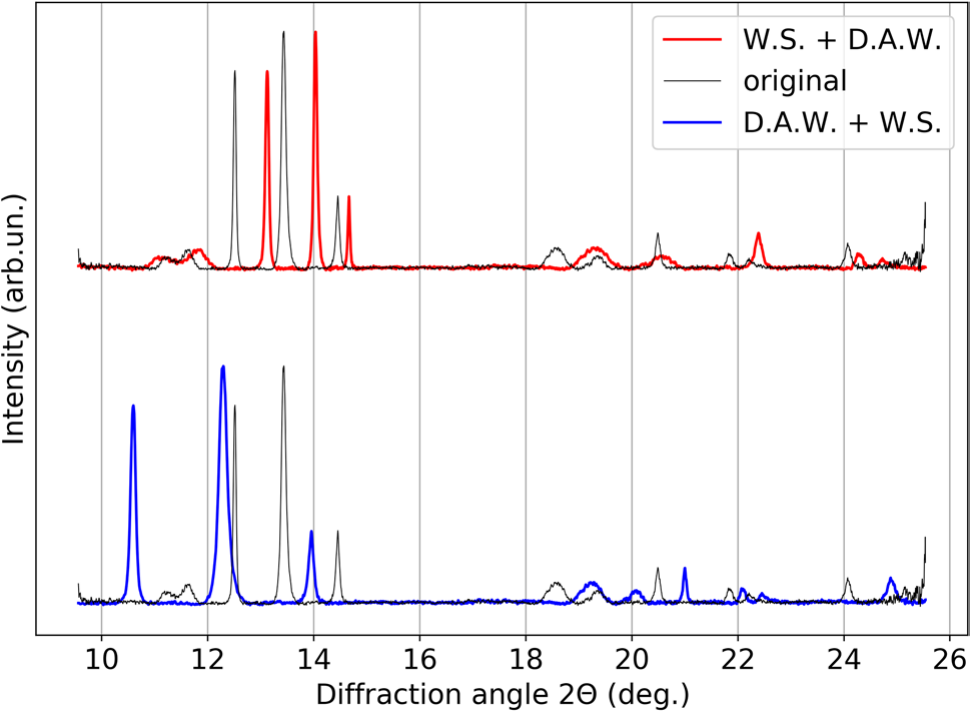
Effect of applying window slice (W.S.) and diffraction angle warping (D.A.W.) data augmentation in a different order. The red line represents the application of W.S. before D.A.W. and the blue line represents the opposite order.

In addition, we found that the order in which data augmentation techniques are applied also affects the results, with different orders leading to different enhanced data, and also because of the inherent randomness of each data enhancement technique. In Fig. 9, we use the combination of window slicing and time warping (Fig. 9), as an example to illustrate the potential importance of the order of applying different data augmentation techniques. The window slicing augmentation randomly removes only the edges of the spectra, while the diffraction angle warping changes the full distribution of peaks in a non-linear way. By applying the window slicing first, the edges are removed, and the remainder of the distribution is warped. It is rare in the given data, that the peaks relevant for the phase transition appear in the edge of the distribution and hence this information is rarely lost. If, on the other hand, the diffraction angle warping is applied first, relevant peaks may be warped to appear in the edges of the distributions, which may be removed when window slicing is applied. In this case, relevant information required for the phase transition identification is removed from the data and the encoder can map the augmented data to a different representation.

This experiment illustrates that data augmentations play an important role in self-supervised models^35,36^. As it is domain-specific, it must be customized for data sets from different research areas. Once the most appropriate data augmentation techniques are identified, the ability to automatically classify the data can be effectively improved.

## Discussion

From the above experimental results, it can be concluded that the three networks proposed in this paper are effective in constructing data representations that can greatly improve the automation of the classifications of spectral data, and in particular the detection of phase transitions. We attribute the success of the models, consistent with the results of the ablation study, to appropriate data augmentations and pretext tasks. In fact, self-supervised learning critically relies on augmentations, which should be tailored for the scientific case object of investigation. The ones applied in this study retain physically meaningful information while simulating other plausible experimental effects. Thus, compared to traditional unsupervised clustering algorithms which require manual tuning of parameters for each dataset, self-supervised models allow the classification process to be automated once a minimal amount of labels is available.

In SpecMoco-Net, the learning process is primarily based on exploiting redundancies in the data, rather than learning to perform inference tasks based on the data itself. In addition, SpecMoco-Net is based on the instance-instance discrimination task, which cannot explicitly exploit data information at different scales, such as the global dependencies across diffraction angle dimension. Furthermore, in practice, self-supervised contrastive learning benefits from a large number of negative samples to extract meaningful representations, and while SpecMoco-Net allows a large and consistent dynamic dictionary, in our case we do not have enough spectral training examples, which may be another important reason why SpecMoco-Net performed slightly worse than SpecRR-Net and SpecRRMoco-Net in our case of study.

Networks based on relational reasoning learning can be viewed as simultaneously learning deep embeddings and non-linear metrics (similarity functions)^42^. In SpecRR-Net and SpecRRMoco-Net, three relational reasoning modules are designed to capture the underlying dependencies from multiple dimensions and at different scales to build useful representations. Moreover, comparison with SpecSelfTime shows that our proposed external-variable relational reasoning module can improve the performance of models by addressing the dependencies of diffraction spectra on pressure values, in this particular application. Relative to the pretext task based on contrastive learning, the relational reasoning-based pretext tasks impose more supervision on the network using easily accessible sources of information. In the process of reasoning about the relations between spectral entities, irrelevant and noisy features are neglected, and non-obvious properties can be focused on, thereby gaining new knowledge. Furthermore, the difference in the structure of the two methods may also lead to some differences in the way of updating model parameters. Ablation studies on structural differences are necessary and interesting for further research, which is left to future work.

SpecRRMoco-Net benefits from both relational reasoning learning and contrastive learning, and shows better results than SpecRR-Net and SpecMoco-Net alone with the current hyperparameters and training settings, it combines SpecRR-Net and SpecMoco-Net therefore providing a flexible framework that can potentially fit a broader set of use cases. The success of each pre-text task in SpecRRMoco-Net drives the update of the encoder model, improving its feature representation ability while increasing the robustness and generality of the encoder network. Importantly, although these models are proposed for classification applications on spectral data, the architectures are general and can be easily extended to 1D time series data and various other types of data, such as image classification.

Further evaluation and interpretation of the model are given in SM-5 of the supplementary material.

## Conclusions

In this paper we propose three self-supervised frameworks to classify 1D spectral data using a minimal amount of labeled data, and we validate their accuracy using x-ray diffraction data of samples showing phase transitions. These frameworks are based on relational reasoning (SpecRR-Net), contrastive learning (SpecMoco-Net) or a linear combination of the two (SpecRRMoco-Net). They are capable of learning discriminative features and building effective representations, therefore greatly reducing the number of labels required, making a step towards automating the spectral classification process. Among them, SpecRRMoco-Net shows superior performance by benefiting from contrastive learning and relational inference learning. Moreover, as a consequence of the reduced number of labels, scientist’s time is greatly optimized. In order to account for the relation between spectra collected along some external variable, we extend the relational reasoning-based method to explicitly include it. In this work, we demonstrate the importance of a proper choice of data augmentations, which must be tailored for the specific case of study to ensure the retention of scientifically meaningful information. In particular, we discuss and validate augmentations relevant to the case study discussed, and we show that the three methods introduced are effective in detecting phase transitions. This is the case even when data for which no labels are available are used, which demonstrates good ability of the approaches. We furthermore compare the three frameworks with state-of-the-art unsupervised methods.

After an initial training step, the methods proposed here can be used to accurately and automatically screen collected data, even in real-time at high-repetition rate facilities given the inference speed, so to provide a better understanding of the experiment and therefore enable the most effective real-time planning.

In future research, we will further validate our self-supervised classification models on spectral data collected from different experiments and spectroscopy techniques. In addition, we plan to work on an automated way of optimizing hyperparameter settings, training strategies and augmentations.

## Supplementary Information

Supplementary Information.

## Data Availability

The data that support the findings of this study are available from the corresponding author upon reasonable request.
