# Supplementary material for: Application of self-supervised approaches to the classification of X-ray diffraction spectra during phase transitions
Source: Sci Rep. 2023 Jun 9;13:9370. doi: 10.1038/s41598-023-36456-y (PMC10256752; doi:10.1038/s41598-023-36456-y)
Supplement: Supplementary file 1 — Supplementary Information. [file 41598_2023_36456_MOESM1_ESM.docx]

**Supplementary Materials**

**Application of self-supervised approaches to the classification of X-ray diffraction spectra during phase transitions**

**Yue Sun^1,2*^, Sandor Brockhauser^1,3^, Péter Hegedűs^1*^, Christian Plückthun^2,4^, Luca Gelisio^2^, and Danilo Enoque Ferreira de Lima^2*^**

^1^ Software Engineering Department, Institute of Informatics, University of Szeged, Dugonics tér 13,

6720 Szeged, Hungary;

^2^ European XFEL GmbH, Holzkoppel 4, 22869 Schenefeld, Germany;

^3^ Center for Materials Science Data, Humboldt-Universität zu Berlin, Zum Großen Windkanal 2, 12489 Berlin, Germany;

^4^ Deutsches Elektronen-Synchrotron (DESY), Hamburg 22607, Germany

**SM-1. Supplementary Methods**

Figure S1 illustrates the shared feature extraction backbone model $f_{q}$, which is based on Conv SC attention model from Ref.^9^.


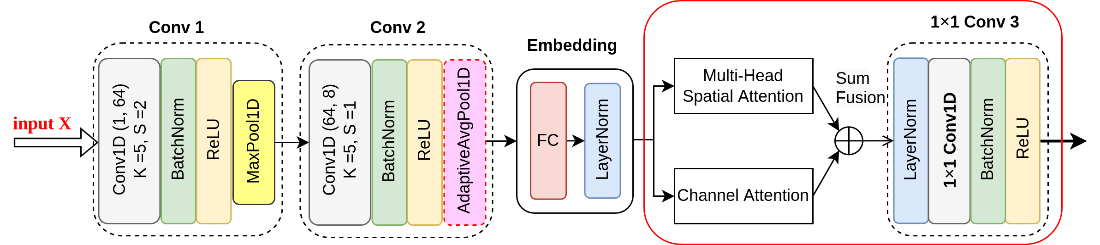


Figure S1. Backbone encoder network based on Conv SC attention model.

**Inter-Sample Relational Reasoning.** In the inter-sample relational reasoning module, the encoded representation $z_{i}^{\left( k \right)}=f_{q}\left( x_{i}^{\left( k \right)} \right)$ can be extracted for the augmented input $x_{i}^{\left( k \right)}$ (k-th augmentation of $x_{i}$ ) after the forward pass of the backbone encoder $f_{q}\left( \cdot\right)$. A positive relational representation pair $\left[ z_{i}^{\left( k1 \right)},z_{i}^{\left( k2 \right)} \right]$ is constructed by aggregating together representations of different views of the same spectra, while a negative representation pair $\left[ z_{i}^{\left( k1 \right)},z_{j}^{\left( k2 \right)} \right]$ is sampled from representations of two randomly paired different spectra, where $\left[ \cdot\right]$ denotes the vector concatenation operation. These two types of relational representation pairs are distinguished by an inter-sample relational reasoning head $r_{\alpha}\left( \cdot\right)$. According to the combination strategy in Ref.^1^, within a minibatch of size B, the total number of pairs $P=B\left( K^{2}-K \right)$. Finally, for a given representation pair $\left[ z_{i},z_{j} \right]$, a relation score $s_{i}^{inter}=r_{\alpha}\left( \left[ z_{i},z_{j} \right] \right)$ can be predicted, whose corresponding target $y_{i}^{inter}$ is equal to 1 for a positive relation pair and 0 for a negative relation pair. This pretext task is trained by minimizing the binary cross-entropy loss $L_{Inter}$

|  | $L_{Inter}=-\frac{1}{P}\sum_{i=0}^{P-1} y_{i}^{inter}\cdot\log\left( s_{i}^{inter} \right)+\left( 1-y_{i}^{inter} \right)\cdot\log\left( 1-s_{i}^{inter} \right)$ | (1). |
| --- | --- | --- |

In this module, we benefit from large number of relation pairs.

**Intra-Sample Relational Reasoning.** Formally, given any augmented spectral curve $x_{i}^{A}$ with $N$ data points, we randomly sample two spectral segments of length L, one starting from diffraction angle $p1$and the other starting from diffraction angle $p2$, denoted $x_{i,1}$ and $x_{i,2}$, as shown in Fig. S2 (a). After propagating through $f_{q}\left( \cdot\right)$, their corresponding representations can be extracted. These are denoted as $z_{i,t1}=f_{q}\left( x_{i,1} \right)$ and $z_{i,t2}=f_{q}\left( x_{i,2} \right)$. Based on this, the intra-relational representation pair is constructed as $\left[ z_{i,t1},z_{i,t2} \right]$ and input to the intra-sample relation reasoning head $r_{\beta}\left( \cdot\right)$ (see Fig. S2 (b)) to reason their spatial relation score $s_{i,c}=r_{\beta}\left( \left[ z_{i,t1},z_{i,t2} \right] \right)$, where $c$ is the class index in the range $\left[ 0, C \right)$ ($C$ is the number of classes). The intra-sample relationship between these two spectral pieces is based on their spatial distance along the diffraction angle dimension, defined as $d_{p1,p2}=\left| p1-p2 \right|$, which is based on the absolute distance between their starting points. We define the number of intra-sample relation types $C=5$, the spectral pieces length $L = 0.2N$, and the distance threshold $D= L/2$. Then the target intra-sample relation label $y_{i}^{intra}$ is assigned by the equation $y_{i}^{intra}= \min\left( floor\left( \frac{d_{p1,p2}}{D} \right),C-1 \right)$. In this way, a multi-class classification task is formulated. The intra-sample relational reasoning loss $L_{Intra}$ is defined as follows:

|  | $L_{Intra}=-\frac{1}{B}\sum_{i=0}^{B-1} \log\frac{\exp\left( s_{i,y_{i}^{intra}} \right)}{\Sigma_{c=0}^{C-1}\exp\left( s_{i,c} \right)}$ | (2), |
| --- | --- | --- |

Figure S2 illustrates the intra-sample relational reasoning module.

(a)
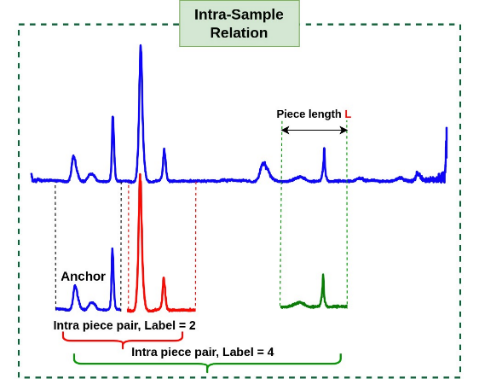
 (b)
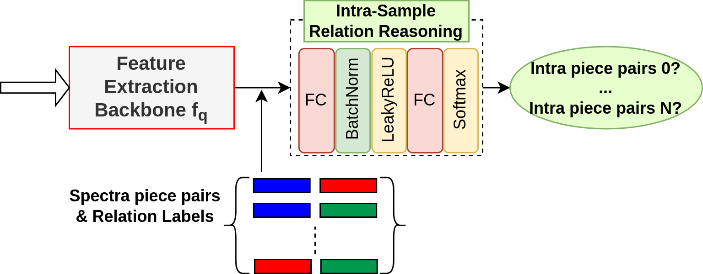


Figure S2. (a) Sampled spectral piece pairs. (b) Intra-Sample relationship and the relation head network.

In the Inter-Sample and Intra-Sample relational reasoning modules, a nonlinear projection head is used.

**Self-Supervised contrastive module.** As can be seen from Fig. S3, there are two structurally identical encoder networks in SpecMoco-Net, i.e., the encoder $f_{q}$ followed by the contrastive projection head $r_{q}$and the momentum encoder $f_{k}$ followed by the projection head $r_{k}.$ In this architecture, the momentum encoder $f_{k}$ is adopted and updated using the momentum update^2^ at each iteration. Furthermore, as in Ref.^2^, Shuffled Batch Normalization (BN)^3^ instead of BN is applied during training. After the contrast pretext task is completed, the contrast head is discarded.


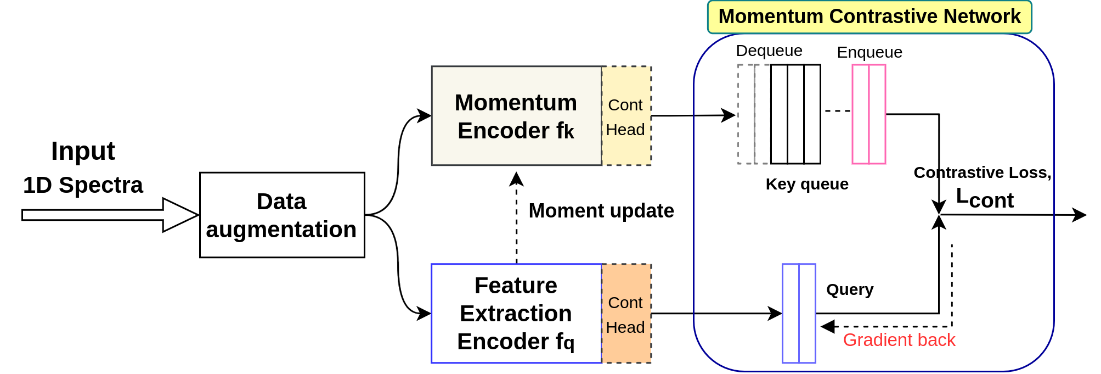


Figure S3. Illustration of SpecMoco-Net.

**SM-2. Supplementary Implementation Details.**

Figure S4 shows the training details of the first stage of SpecRRMoco-Net, where the training accuracies of relational reasoning pretext tasks (Fig. S4 (a)) increase with the decrease of the corresponding of relational losses (Fig. S4 (b)), and the contrastive loss (the last column of Fig. S4 (a)) decreases in the same trend.

(a)
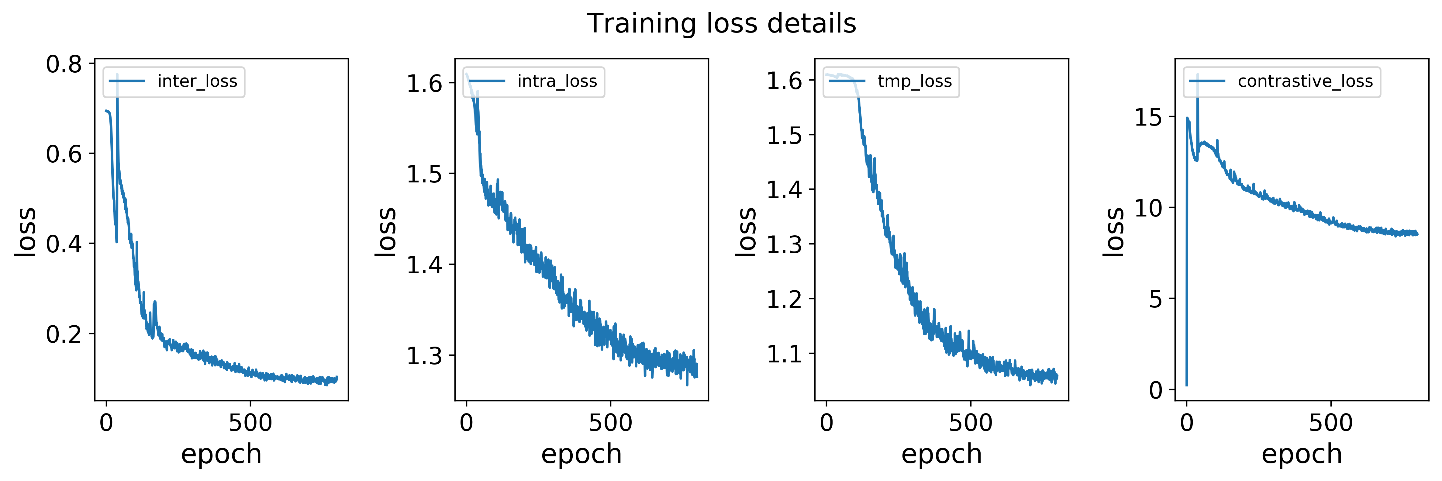


(b)
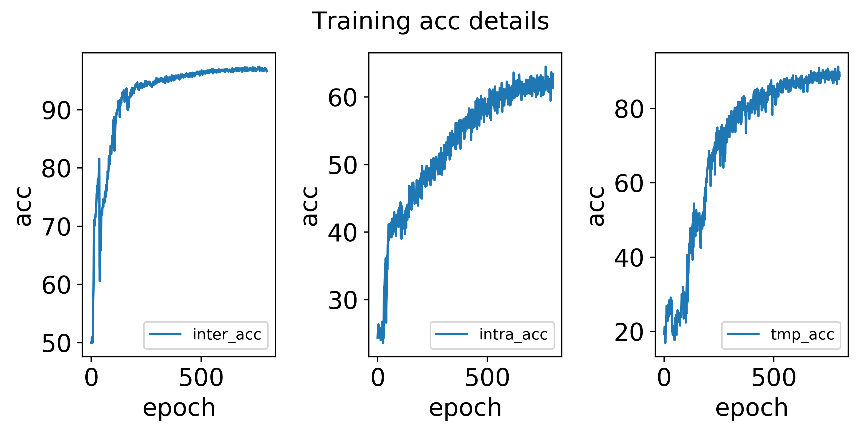


Figure S4. (a) Training loss and (b) training accuracy at different epochs for each pretext task in SpecRRMoco-Net during the first stage training. In SpecRRMoco-Net, the encoder is trained under the supervision of inter-sample, intra-sample, and external-variable relational reasoning heads, as well as SpecMoco contrastive head. Notably, the instance discrimination pretext task based on self-contrastive learning (SpecMoco-Net) has no accuracy-based metric, it is based on a similarity measure of the learned representations rather than a classification objective. Therefore, we show the distribution of loss values for four loss function components and classification accuracy of three relational reasoning-based pretext tasks.

Among the 42 representative scattering curves, 22 belong to the ‘before phase transition’ category, 5 belong to the ‘during phase transition’ category, and 15 belong to the ‘after phase transition’ category. In the linear evaluation stage, we applied a stratified random split to the imbalanced labeled data with a train/validation split ratio of 66.7%/33.3% to ensure at least two scattering curves corresponding to each phase. The best model on the validation dataset was used for testing, updated only if higher validation accuracy was achieved in the process. Typically, after training for several epochs, the model can achieve high classification accuracy. This illustrates the high quality of the encoder network, indicating that the backbone encoder has extracted useful representations from the spectral data.

**SM-3. Supplementary Results**

|  | **Model** | **Fe** | | | | **FeO** | | | | | |
| --- | --- | --- | --- | --- | --- | --- | --- | --- | --- | --- | --- |
|  |  | **Precision**  **(C0/C2)** | | **Recall**  **(C0/C2)** | | **Precision**  **(C0/C1/C2)** | | | **Recall**  **(C0/C1/C2)** | | |
| 2.8% labels | SpecSelfTime | 99.5±0.2 | 98.2±0.7 | 95.2±2.2 | 99.58±0.1 | 76.8±5.6 | 45.7±5.9 | 86.5±1.5 | 81.0±6.0 | 45.4±3.7 | 86.9±5.4 |
|  | SpecRR-Net | 97.4±0. 5 | 99.8±0.3 | 99.4±1.0 | 99.0±0.2 | 89.1±12.1 | 61.6±24.1 | 96.8±3.3 | 84.6±10.9 | 71.5±24.4 | 94.7±3.7 |
|  | SpecMoco | 94.0±3.9 | 99.9±0.3 | 99.7±0.8 | 97.3±2.2 | 90.0±9.2 | 82.8±16.6 | 97.3±1.4 | 97.2±3.3 | 65.8±12.9 | 95.6±5.2 |
|  | SpecRRMoco | **98.7**±**0.5** | **99.9**±**0.2** | **99.6**±**0.6** | **99.5**±**0.2** | **98.5**±**2.8** | **83.5±16.2** | **97.2±2.8** | **97.0±3.0** | **83.1±18.3** | **98.3±1.8** |
| 10% labels | SpecSelfTime | 99.4±0.4 | 98.2±0.4 | 95.7±1.0 | 99.8±0.2 | 82.3±9.9 | 31.4±6.1 | 84.8±1.9 | 63.5±10.1 | 43.3±5.7 | 90.4±7.0 |
|  | SpecRR-Net | 96.5±0.4 | 100.0±0.0 | 99.7±0.5 | 98.7±0.2 | 97.7±4.0 | 73.4±3.2 | 99.8±0.2 | 93.4±1.5 | 97.6±1.5 | 96.9±1.7 |
|  | SpecMoco | 97.4±1.1 | 100.0±0.0 | 100±0.0 | 99.1±0.4 | 94.9±2.1 | 83.4±5.2 | 95.2±0.6 | 94.9±1.7 | 66.2±6.2 | 97.1±0.9 |
|  | SpecRRMoco | **98.3**±**0.4** | **100.0**±**0.0** | **99.8**±**0.5** | **99.5**±**0.1** | **97.5±1.0** | **80.3±10.4** | **99.4±1.9** | **98.0±1.3** | **91.9±13.1** | **97.4±0.6** |

Table S 1. Per-class precision and recall for the Fe datasets and the FeO datasets using different self-supervised methods. For each method, the classification results are reported with amounts of labels corresponding to either 2.8% or 10% of the total collected data. C0/C1/C2 correspond to the categories before, during and after the phase transition, respectively.

| **c** | **Fe** | | | | **FeO** | | | | | |
| --- | --- | --- | --- | --- | --- | --- | --- | --- | --- | --- |
|  | **Precision**  **(C0/C2)** | | **Recall**  **(C0/C2)** | | **Precision**  **(C0/C1/C2)** | | | **Recall**  **(C0/C1/C2)** | | |
| 0.001 | 96.3±3.1 | 99.1±0.5 | 95.9±4.5 | 98.8±0.1 | 97.5±7.4 | 66.0±19.0 | 94.8±2.6 | 84.3±10.2 | 66.1±17.5 | 99.1±2.7 |
| 0.01 | **98.7±0.5** | **99.9**±**0.2** | **99.6±0.6** | **99.5±0.2** | **98.5±2.8** | **83.5±16.2** | **97.2±2.8** | **97±3.0** | **83.1±18.3** | **98.3±1.8** |
| 0.1 | **98.6±0.4** | **99.8±0.3** | **98±3.3** | **99.5±0.1** | **95.9±3.1** | **94.7±6.0** | **98.1±1.1** | **97±4.5** | **82.2±9** | **99.6±0.9** |
| 1 | 96.6±2.4 | 99.7±1.6 | 94.7±8.0 | 98.7±1.1 | 98.8±1.5 | 61.3±11.6 | 98.3±1.8 | 98.8±1.5 | 61.3±11.6 | 98.3±1.8 |
| 0  (SpecRR-Net) | 97.4±0. 5 | 99.8±0.3 | 99.4±1.0 | 99.0±0.2 | 89.1±12.1 | 61.6±24.1 | 96.8±3.3 | 84.6±10.9 | 71.5±24.4 | 94.7±3.7 |
| Inf  (SpecMoco-Net) | 94.0±3.9 | 99.9±0.3 | 99.7±0.8 | 97.3±2.2 | 90.0±9.2 | 82.8±16.6 | 97.3±1.4 | 97.2±3.3 | 65.8±12.9 | 95.6±5.2 |

Table S 2. Ablation study of the coefficient $c$ in the loss function of RRMoco-Net. Per-class precision and recall for Fe and FeO are reported as average and standard deviations over 20 runs. For each method, the classification results are reported with amounts of labels corresponding to 2.8% of the total collected data. C0/C1/C2 correspond to the categories before, during and after the phase transition, respectively.

(a)
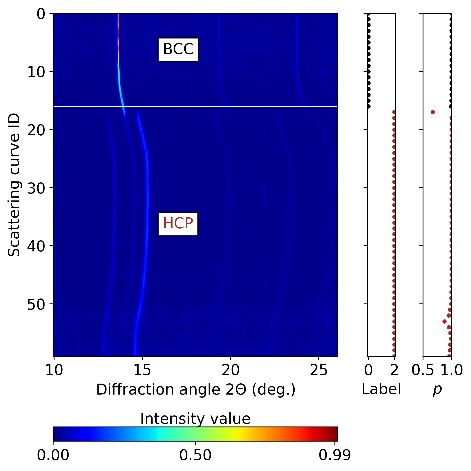
 (b)
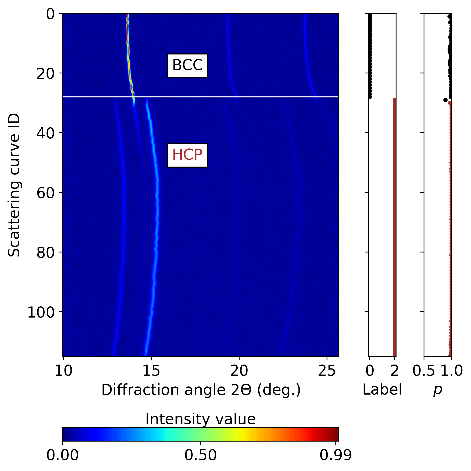
 (c)
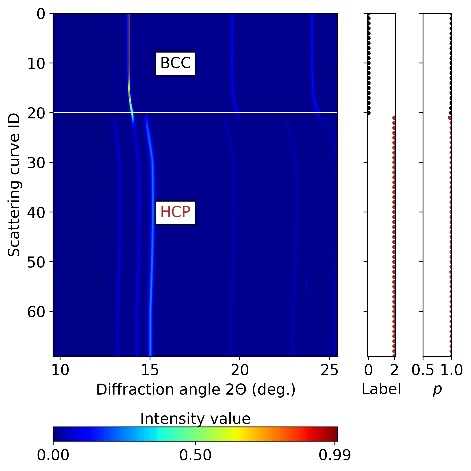


(d)
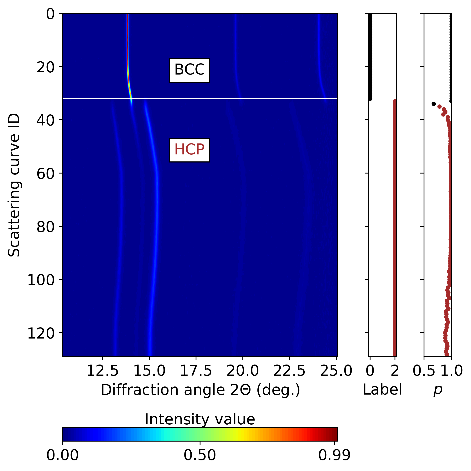
 (e)
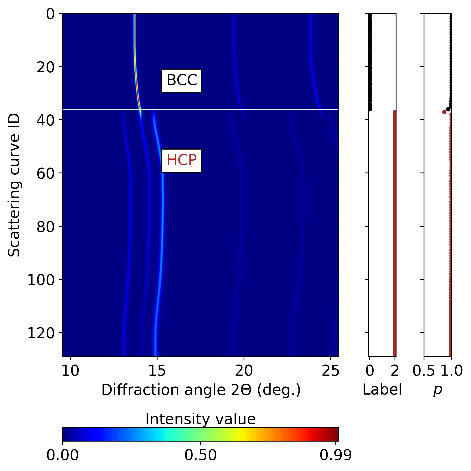
 (f)
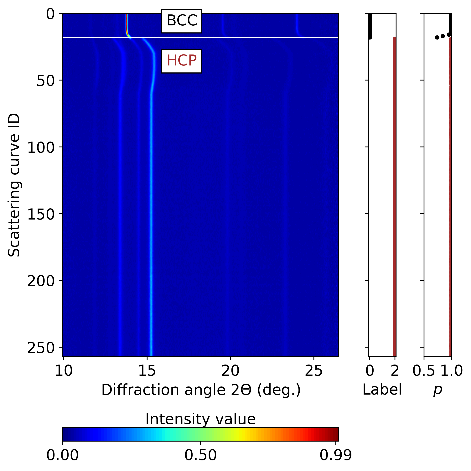


Figure S5. Classification results of SpecRRMoco-Net on the experimental spectral data with 2.8% labeled data. Black corresponds to data sets collected before the phase transition, magenta during, and brown after. Horizontal lines in a contour map indicate the onset or end of a phase transition. The label predicted by SpecRRMoco-Net is also reported, together with the associated probability $p$. Each column corresponds to a different data set. Results on the full (a) D1 dataset, (b) D2 dataset, (c) D3 dataset, (d) D4 dataset, (e) D5 dataset, (f) D7 dataset.

As can be seen in Fig. S6, the predicted class labels of the D8 spectra data sets vary inconsistently in SpecMoco-Net, and no clear boundaries can be detected. In SpecSelfTime, a considerable number of class labels on the D8 dataset were incorrectly predicted and some predicted class labels in D8 dataset jump inconsistently around the ‘during phase transition’ region. In SpecRR-Net, the spectra data sets are well classified.

(a)
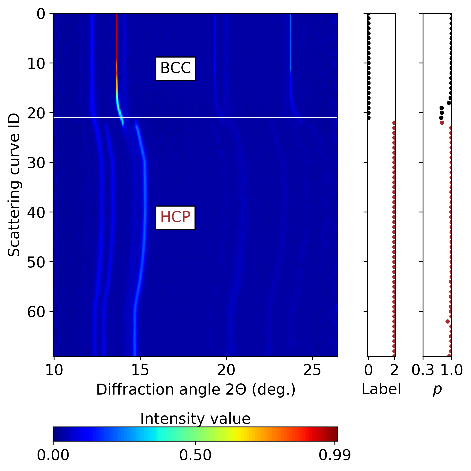

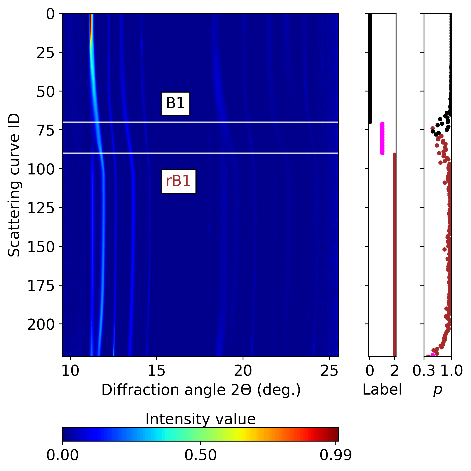

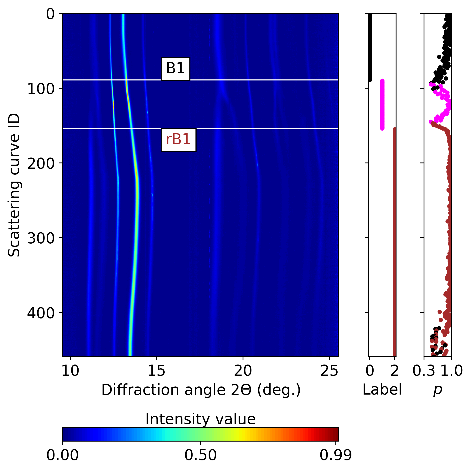


(b)
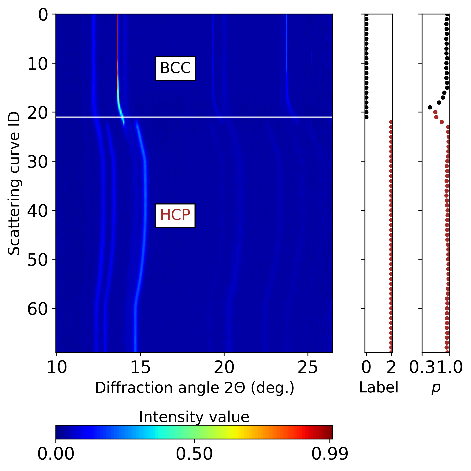

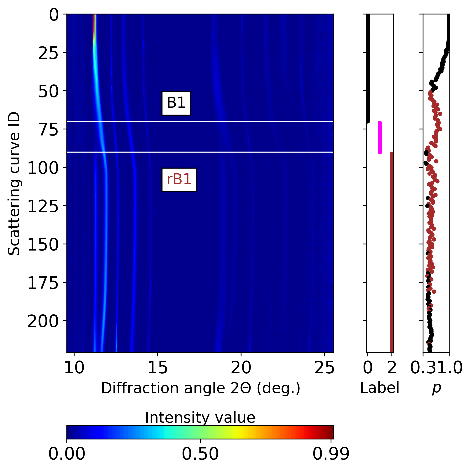

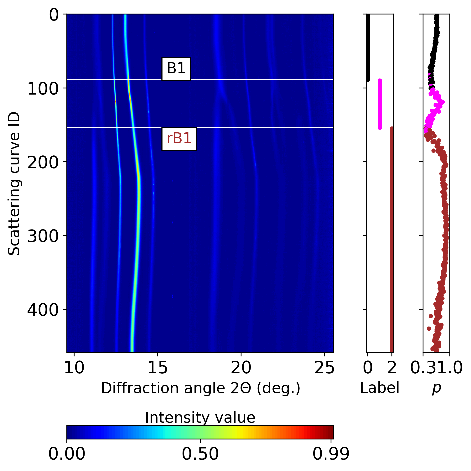


(c)
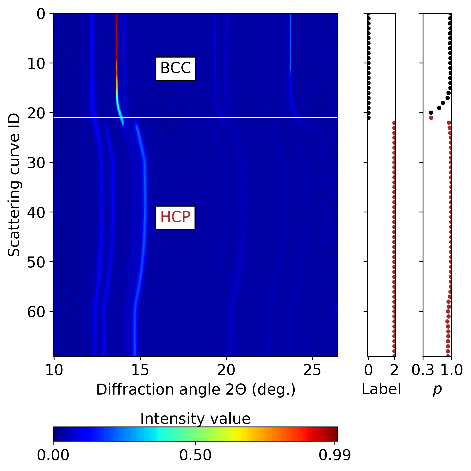

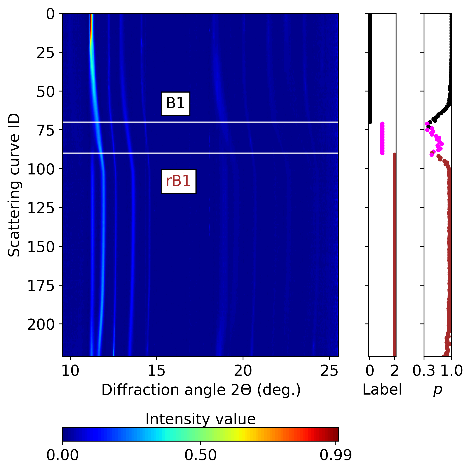

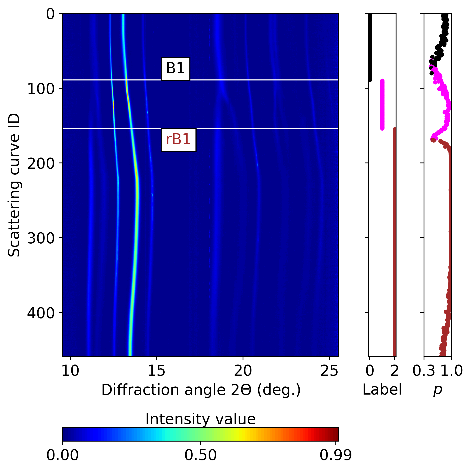


Figure S6. (a) Classification results of the SpecMoco-Net on the experimental spectral data (D6, D8, and D9) with 2.8% labeled data. (b) Classification results of SpecSelfTime on the experimental spectral data (D6, D8, and D9) with 2.8% labeled data. We can find that the phase transition region cannot be detected in the D8 dataset. This result indicates that the generalization ability of the model is low. (c) Classification results of SpecRR-Net (D6, D8, and D9) on the experimental spectral data sets.

Figure S7 (a), (b), and (c) show the representations learned from the SpecMoco-Net, SpecRR-Net, and SpecSelfTime models, respectively. In all cases, the class labels are ground truth. It can be seen from these visualizations that these self-supervised models have a strong clustering ability, with spectra belonging to the same class clustered together and those of different classes far apart.

(a)
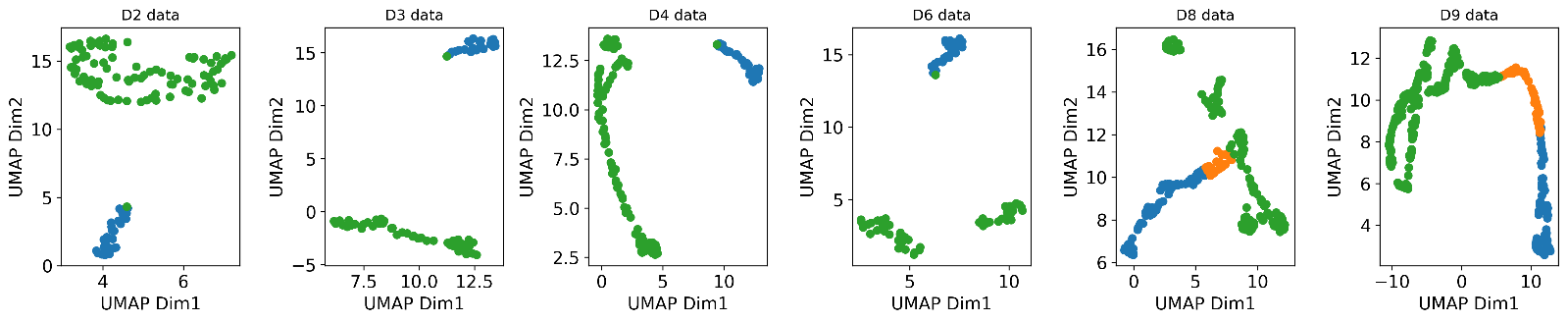


(b)
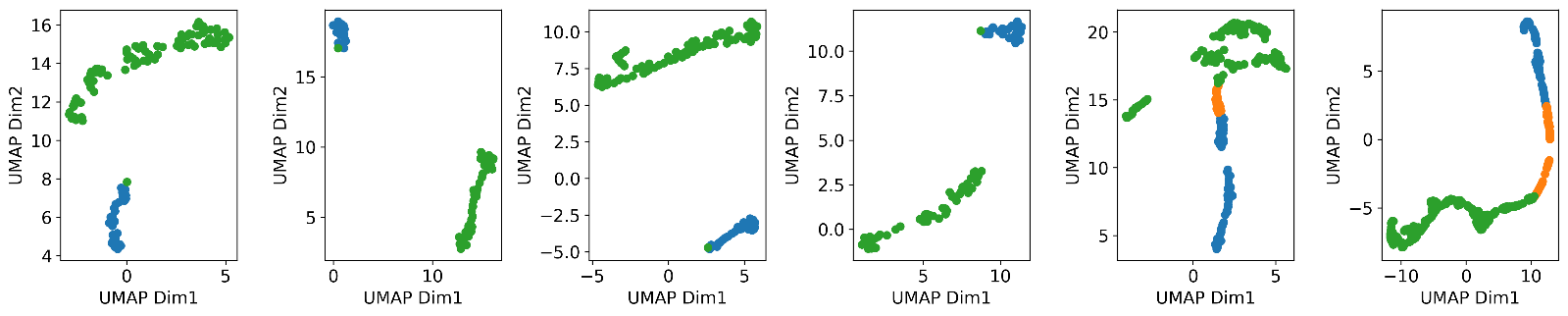


(c)
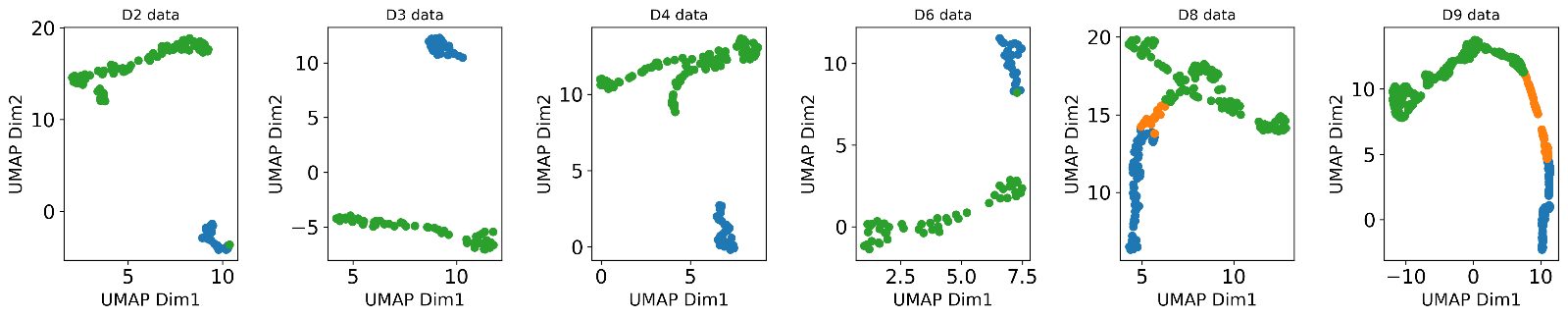


Figure S7. UMAP visualization of learned representations in the embedding space after different backbone encoders. (a) UMAP visualization of the embedded features after SpecMoco-Net encoder. (b) UMAP visualization of the embedded features after SpecRR-Net encoder. (c) UMAP visualization of the embedded features after SpecSelfTime. For all the visualization results, the class labels are ground truth.

The predicted labels were obtained by applying spectral clustering methods to the original data or to the representations learned from different self-supervised encoders. the Rand index values were calculated between the ground truth labels and the predicted labels. In spectral clustering method applied to all the models, the number of clusters was set to 3 for the Fe data sets, and 2 for the FeO data sets. In addition, in spectral clustering, a K-means strategy or Discretization^6^ is applied to assign labels and the affinity matrix is constructed by computing the nearest neighbor graph or radial basis function (RBF) kernel. The mean and standard deviation values are calculated from different combinations of these parameters (four groups).

| **Model** | **Average** | |
| --- | --- | --- |
|  | **Fe** | **FeO** |
| Spectral Clustering (SC) | 0.86±0.08 | 0.76±0.03 |
| SpecSelfTime + SC | 0.96±0.03 | 0.77±0.04 |
| SpecRRMoco (c 0.01) + SC | 0.97±0.02 | 0.85±0.04 |
| SpecRR-Net + SC | 0.98±0.00 | 0.85±0.05 |
| SpecMoco + SC | 0.91±0.09 | 0.85±0.05 |

**Table S 3.** The Rand Index values between the ground truth labels and the predicted labels of different methods for each data set. The first row corresponds to the Rand Index between the ground truth labels and the predicted labels obtained by applying Spectral Clustering to the original data, while the following rows show the values between the ground truth labels and the predicted labels obtained by applying Spectral Clustering to the latent representation produced after the encoder trained with the respective self-supervised learning techniques. Results are reported for the average results for the Fe dataset group, and the FeO dataset group.

**SM-4. Parameters in data augmentation**

**Magnitude warping:**  This data augmentation is achieved by multiplying the original data by a curve created by cubic spline with 4 knots. The amplitude of the curve is sampled from a normal distribution with a mean of 1 and a standard deviation of 0.3;

**Diffraction Angle warping**: In this augmentation, the diffraction angle dimension is warped by a by a cubic spline with 8 knots. The cubic spline is the result of a fit of a curve produced from a normal distribution with a mean of 1 and a standard deviation of 0.2;

**Window slicing:** This augmentation is performed by cropping out 80% of the contiguous spectrum of the original spectrum and then interpolating it to the original length. The starting point of the spectrum is obtained by randomly sampling between 0 and 20% of the original spectrum.

**Jittering:** It is achieved by adding noise sampled from a normal distribution with a mean of 0 and a standard deviation of 0.1;

**Scaling:** It is achieved by multiplying by a constant sampled from a normal distribution with a mean of 1 and a standard deviation of 0.4.

**SM-5. Model Interpretation and Further evaluation**

To interpret these self-supervised models, taking SpecRRMoco-Net model as an example, we visualized the learned representations of some example spectra data sets (D4, D8 and slow D9). From the visualization in Fig. S8, we can get that distinct patterns can be learned from the SpecRRMoco encoder network.


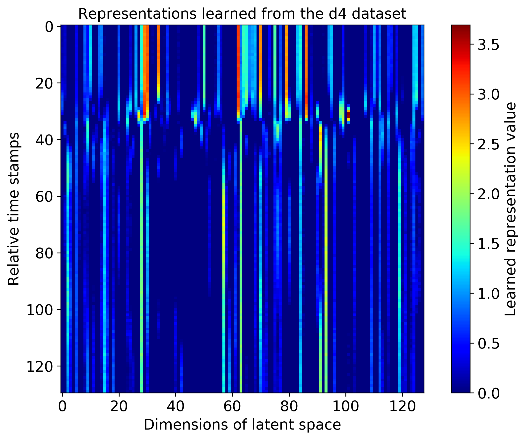

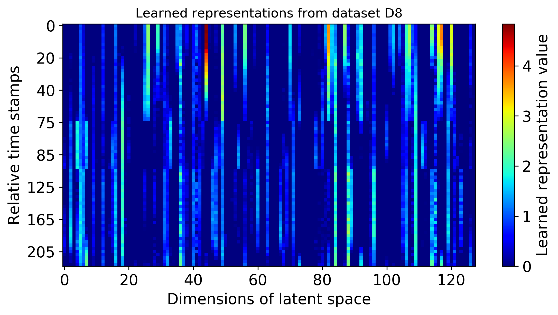

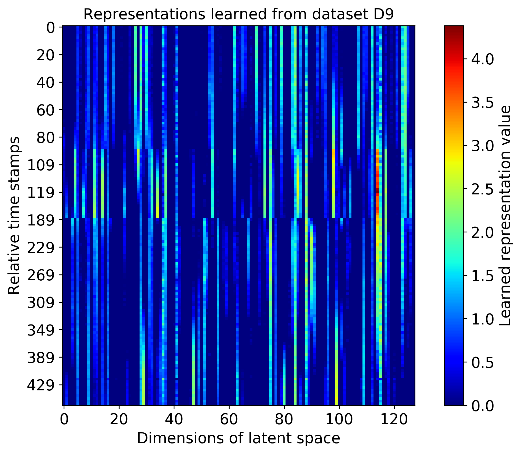


Figure S8. Visualizations of the learned representations on data sets D4, D8, D9, respectively.

We also conducted an experiment to test the performance of SpecRRMoco-Net on noisy data. Noise is collected from a normal (Gaussian) distribution that is 0.5 times the normalized noise. We applied spectral clustering on the learned representations from the encoder network $f_{\theta}\left( \cdot\right)$, so we did not make use of any label information. Compared to applying the spectral clustering method directly to noisy data, we found that our model is more robust than the spectral clustering method.

Taking the D5 dataset as an example, Fig. S9 (a), and (b) show the UMAP visualization of noisy data and its corresponding representation learned by SpecRRMoco-Net, respectively. In both cases, the label is the ground truth. This experiment shows that the SpecRRMoco achieves better classification results on noisy data and has better clustering ability. It also illustrates that this key design principle based on data enhancement techniques makes the self-supervised learning models more robust to input perturbations.

(a)
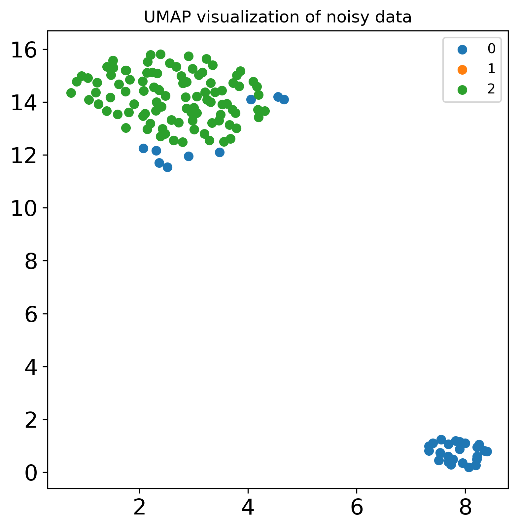
 (b)
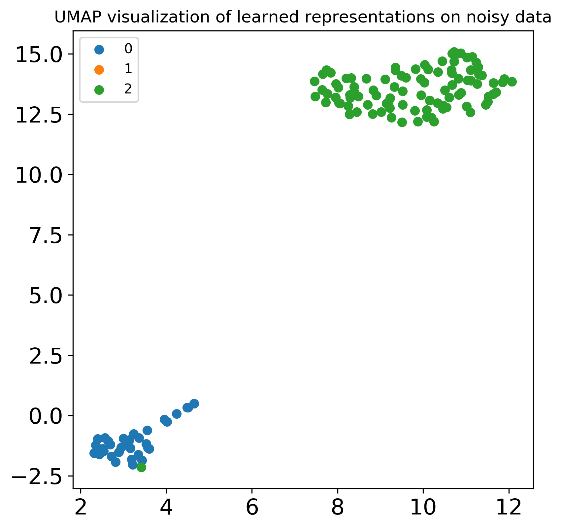


Figure S9**.** Clustering ability on noisy data (D5). (a) UMAP visualization of the noisy example dataset. Class labels are ground truth. (b) UMAP visualization of learned representations of noisy data by the SpecRRMoco encoder. Class labels are ground truth.

**Gradient-based feature importance analysis.** We further interpret SpecRRMoco-Net by gradient-based feature importance analysis, as shown in Fig. S10. Since the gradient can reflect the overall impact of each input feature on the current class prediction^4,5^, in this experiment, we perform a feature importance attribution analysis to further quantify interpretability of the model and evaluate its classification performance. Taking the D9 dataset as an example, three representative spectral curves (belonging to before, during, and after the phase transition, respectively) are shown in Fig. S10(Top row), and Fig. S10(Bottom row) shows the contribution map of the example dataset, which is the cumulative result of gradiences of all spectral curves in the dataset. As can be seen from the feature importance analysis, the attribution map can dynamically focus on the highly separable and physically meaningful features (peaks regions) relevant to classification, while suppressing misleading indistinguishable and noisy features. This further proves the high quality of the SpecRRMoc-Net and the accuracy of the classification model.


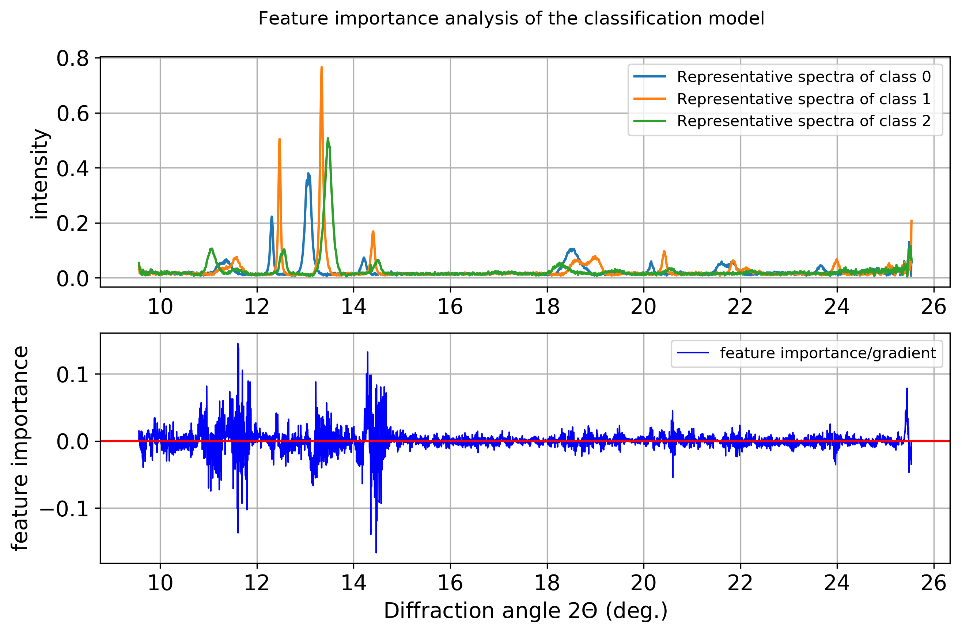


Figure S10. Feature importance analysis of SpecRRMoco-Net on Slow dataset. The feature importance analysis is based on gradient backpropagation. (Top row): Representative spectral curves (before, during, and after phase transition) in the D9 dataset for each class. (Bottom row): Feature importance map on the D9 dataset. Feature importance values are calculated from the sum of the gradients of all spectral curves in the example dataset.

**Supplementary References**

1. Patacchiola, M. and Storkey, A.J. Self-supervised relational reasoning for representation learning. *Advances in Neural Information Processing Systems*. **33**, 4003-4014 (2020).
2. He, K., Fan, H., Wu, Y., Xie, S., & Girshick, R. Momentum contrast for unsupervised visual representation learning. In *Proceedings of the IEEE/CVF conference on computer vision and pattern recognition*. 9729-9738 (2020).
3. Ioffe, S. & Szegedy, C. Batch Normalization: Accelerating Deep Network Training by Reducing Internal Covariate Shift. in *Proceedings of the 32nd International Conference on Machine Learning* 448–456 (PMLR, 2015).
4. Sun, Y., Brockhauser, S. & Hegedűs, P. Comparing End-to-End Machine Learning Methods for Spectra Classification. *Appl. Sci.* **11**, 11520 (2021).
5. Rußwurm, M. & Körner, M. Self-attention for raw optical Satellite Time Series Classification. *ISPRS J. Photogramm. Remote Sens.* **169**, 421–435 (2020).
6. Stella, X. Y., & Shi, J. Multiclass spectral clustering. In *Computer vision, IEEE international conference on* (Vol. 2, pp. 313-313). IEEE Computer Society (2003, October).
